# Cation-Disordered Rock-Salt Lithium Titanium Oxyfluoride Anode Enabling High-Rate Li-Ion Storage Through a 3D Percolation Network

**DOI:** 10.1007/s40820-026-02123-w

**Published:** 2026-03-10

**Authors:** Jing Gao, Minghao Hua, Junze Lu, Yuying Qin, Shuxian Zhang, Qingyu Li, Lidong Yang, Chengxiang Wang, Xiaohang Lin, Yuanwei Sun, Longwei Yin, Rutao Wang

**Affiliations:** 1 Shandong Provincial Key Laboratory of Electrochemical Catalysis and Conversion, School of Materials Science and Engineering, Shandong University, Ji’nan, 250061 People’s Republic of China; 2 School of Traffic Management Engineering, Shandong Police College, Ji’nan, 250014 People’s Republic of China; 3 Department of Industrial and Systems Engineering, The Hong Kong Polytechnic University, Hong Kong, People’s Republic of China; 4 Medical Science and Technology Innovation Center and Electron Microscopy Center, Shandong First Medical University and Shandong Academy of Medical Sciences, Ji’nan, 250117 People’s Republic of China

**Keywords:** Disordered rock-salt materials, Pseudocapacitance, Three-dimensional Li^+^ percolation network, Anode materials, Lithium-ion capacitors

## Abstract

Pseudocapacitive materials employ rapid, non-diffusion-limited faradaic processes to store charge, demonstrating significant potential for fast-charging batteries and supercapacitors. However, the high redox potentials of existing pseudocapacitive anodes substantially lower the overall cell voltage and energy density. Here, a cation-disordered rock-salt lithium titanium oxyfluoride (DRX-Li_x_TiOF_2_, 0 < x < 2) is reported to reversibly accommodate approximately 1.19 mol of Li^+^ (~ 310 mAh g^−1^) and delivers high-rate performance (exceeding 64.4 C) via pseudocapacitive Li^+^ storage within a low potential window extending down to 0.1 V vs. Li^+^/Li. This pseudocapacitive behavior is characterized by several structural and electrochemical features: the absence of phase transformation during Li^+^ intercalation, quasi-rectangular cyclic voltammetry curves, sloping charge/discharge profiles and a surface-controlled current response. We further reveal that the pseudocapacitive characteristics originate from a three-dimensional percolation network that facilitates fast Li^+^ migration with low energy barriers, enabled by a cation/anion-disordered structure arising from the mixed occupancy of Li/Ti cations and O/F anions. Owing to its low operating potential and high-rate capability, DRX-Li_x_TiOF_2_ allows a lithium-ion capacitor to attain 4.0 V cell voltage and achieve energy and power densities several times higher than those obtained with conventional anodes, such as battery-type Li_4_Ti_5_O_12_ or pseudocapacitive materials like Nb_2_O_5_ and TiO_2_.

**Supplementary Information:**

The online version contains supplementary material available at 10.1007/s40820-026-02123-w.

## Highlights

- A novel low-potential cation-disordered rock-salt lithium titanium oxyfluoride (DRX-Li_x_TiOF_2_) anode synthesized via electrochemically induced transformation enables pseudocapacitive Li^+^ storage extending down to 0.1 V vs. Li^+^/Li and delivers a high reversible capacity of ~ 310 mAh g^−1^ and an ultrahigh rate capability exceeding 64.4 C.
- Monte Carlo simulations reveal that the pseudocapacitive characteristics of DRX-Li_x_TiOF_2_ anode originate from a three-dimensional percolation network that facilitates fast Li^+^ migration with low energy barriers, enabled by a cation/anion-disordered structure arising from the mixed occupancy of Li/Ti cations and O/F anions.
- The lithium-ion capacitor assembled with this DRX-Li_x_TiOF_2_ anode and an activated carbon cathode exhibits exceptional performance: a 4.0 V operating voltage, a high energy density of 197.9 Wh kg^−1^ and an ultrahigh power density of 50,000 W kg^−1^.

**Supplementary Information:**

The online version contains supplementary material available at 10.1007/s40820-026-02123-w.

## Graphical abstract

**Figure:**
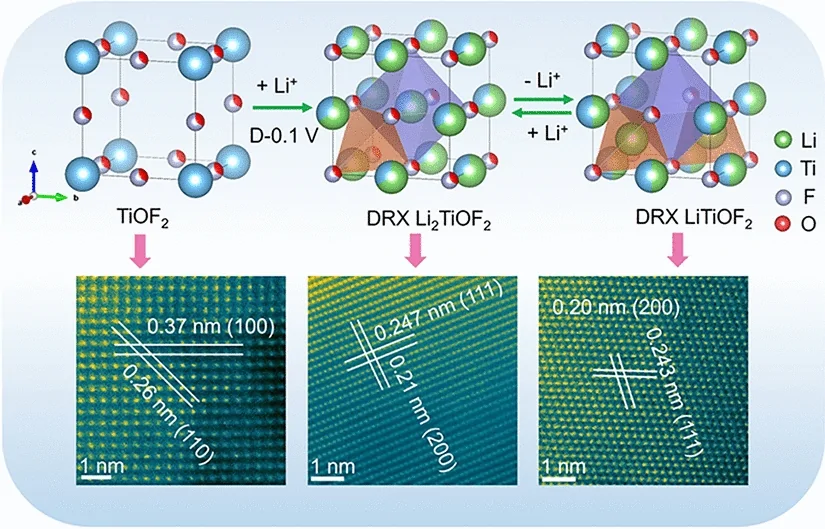

## Introduction

With the rapidly expanding markets for electric vehicles, rail transit and large-scale grid storage, there is a growing demand for electrochemical energy storage (EES) systems capable of fast charging, long cycle life and high energy density [1–4]. At present, commonly used EES technologies such as lithium-ion batteries (LIBs) and supercapacitors (SCs) only partially fulfill these requirements [5, 6]. While LIBs can achieve high energy densities over 300 Wh kg^−1^, they still suffer from slow charge/discharge kinetics and unsatisfactory cycle life [7]. By contrast, SCs provide faster charge/discharge rates and much longer lifetimes, but are restricted by their lower energy density (5 Wh kg^−1^) [8, 9]. The different charge storage properties within LIBs and SCs result from fundamentally different energy storage mechanisms, with battery materials operating through diffusion-limited redox reactions and capacitive materials relying on fast double-layer ions adsorption/desorption. Consequently, there is a compelling need for electrode materials that combine the high capacity of battery-type materials with the high-rate capability of capacitive materials. Recently, more evidences show that pseudocapacitive materials can meet this requirement because they deliver large amounts of energy of energy through battery-like reactions at fast-charging rates [10–12].

The charge storage mechanism in pseudocapacitive materials fundamentally sets them apart from battery-type materials, leading to distinctly different electrochemical characteristics [11]. The charge storage of pseudocapacitive materials occurs at surface, near-surface regions, channels or layers; hence, the kineties is surface-controlled and even highly comparable to that of capacitive materials [13]. Perhaps more important, pseudocapacitive materials undergo no phase transformation during the operation, thereby exhibiting intrinsic structural stability and long cycle life. Pseudocapacitive materials typically exhibit capacitor-like electrochemical responses. Key features include: (i) quasi-rectangular cyclic voltammetry (CV) curves; (ii) sloping charge/discharge profiles with minimal voltage hysteresis; and (iii) a nearly vertical line in the Nyquist plot, corresponding to a phase angle close to 90° [10]. Typical early pseudocapacitive materials are transitional metal oxides such as RuO_2_ [14] and MnO_2_ [15], as well as nitrides (e.g., VN [16]) which store charge via surface redox reactions in aqueous electrolytes. Given that the thermodynamic limit of water, SCs based on these pseudocapacitive materials exhibit lower cell voltage (< 2.0 V) and energy density, which do not significantly surpass traditional SCs performance [17]. Thus, research has shifted toward on non-aqueous pseudocapacitive materials, which are expected to replace the graphite anode to fabricate fast-charging LIBs or Li-ion capacitors (LICs) [8, 11]. Non-aqueous pseudocapacitive materials, such as T-Nb_2_O_5_ [18] TiO_2_(B) [19] and Nb_16_W_5_O_55_ [7], possess large, open channels or layered structures, allowing the fast ion diffusion in bulk and accommodating significant amounts of Li^+^ at high rates. However, these pseudocapacitive materials usually feature with higher intercalation potentials (> 1.0 V versus Li^+^/Li). This phenomenon renders both operating voltage and energy density decreasing, which is an undesirable yet often unavoidable trade-off in LIBs and LICs. Nevertheless, pseudocapacitive materials still show considerable promise for developing advanced EES with both high energy and high power.

Here, we present a new pseudocapacitive material, the cation-disordered rock-salt (DRX) Li_x_TiOF_2_ (0 < x < 2), which delivers a high capacity of 310 mAh g^−1^ at 0.1 A g^−1^ while maintaining over 93 mAh g^−1^ at a high rate of 20 A g^−1^ (64.4 C). DRX-Li_x_TiOF_2_ is formed by an electrochemical induced transformation from cubic TiOF_2_. Its pseudocapacitive behavior was determined from structural signatures of no phase change upon lithiation and electrochemical signatures of quasi-rectangular CV profile, linear charge/discharge curve and surface-controlled current response. Monte Carlo (MC) simulations reveal the formation of a unique 3D Li-ion percolation network in DRX-Li_x_TiOF_2_. This network, composed of 0-TM channels (where face-sharing octahedra lack transition metals), is the origin of the observed pseudocapacitive behavior. Additionally, nudged elastic band (NEB) calculation results point out that the Li^+^ migration mainly takes place through a cooperative mechanism enabled by low-energy-barrier tetrahedral–tetrahedral (t–t) and tetrahedral–octahedral–tetrahedral (t–o–t) pathways. Furthermore, LIC based on this DRX-Li_x_TiOF_2_ anode yields high voltage (4.0 V), high energy (197.9 Wh kg^−1^) and high power (50,000 W kg^−1^). This study highlights the importance of exploiting a new type of pseudocapacitive materials to address the requirement from advanced EES systems, characterized by their need for progressive performance in energy, power and cycling life.

## Experimental Section

### Materials

All chemicals are analytical grade and are used directly without any purification. HF (AR, ≥ 99.0%), CH_3_COOH (AR, ≥ 99.9%), tetrabutyl titanate (C_16_H_36_O_4_Ti, AR, ≥ 99.0%) and 1-methyl-2-pyrrolidinone (NMP, AR, ≥ 99.9%) were purchased from Aladdin Chemicals. Polyvinylidene fluoride (PVDF), acetylene black and Li foil are purchased from MTI Corporation. 1 M LiPF_6_ in EC:DMC:EMC = 1:1:1 vol% was purchased from DoDoChem of China.

### Preparation of DRX-Li_x_TiOF_2_ Anode, PDPC Cathode

TiOF_2_ was synthesized using a modified hydrothermal method [20]. 5 mL of HF, 15 mL of C_16_H_36_O_4_Ti and 30 mL of CH_3_COOH were added into a container of polytetrafluoroethylene and stirred for 0.5 h. It was then heated at 200 °C for 24 h. The product was washed several times with deionized water and ethanol, and then dried in a vacuum oven at 60 °C for 12 h.

For the fabrication of the DRX-Li_x_TiOF_2_ anode, as-prepared TiOF_2_, acetylene black and PVDF with a 6:3:1 mass ratio in NMP were mixed with a mortar. The well-mixed slurry is uniformly coated onto the copper foil. Then the film was heated at 80 °C for 8 h in the vacuum oven. The film was punched into 14-mm-diameter circular electrodes with an active material mass loading of approximately 1 mg. For the lithium half-cell, the electrodes were assembled in 2032-type coin cells with Li foil, a Celgard 2400 separator and 1 M LiPF_6_ in EC:DMC:EMC (1:1:1 by volume, 50 uL) within an Ar-filled glove box. The O_2_ and H_2_O levels inside the box were stably maintained below 0.1 ppm. DRX-Li_x_TiOF_2_ was synthesized by electrochemical lithiation of TiOF_2_ to 0.1 V. The cells were cycled under a potentials window of 0.1–2.0 V (Li^+^/Li).

Using polyphenylene derived porous carbon (PDPC) as cathode material for LICs. The PDPC was synthesized according to our previous report [21]. The 80 wt% PDPC, 10 wt% acetylene black and 10 wt% polytetrafluoroethylene (PTFE) were mixed and then were rolled into thin sheets. After heated at 120 °C for 10 h, it was stored in a desiccator.

### Structural Characterization

The morphology and structure of as-prepared TiOF_2_ and DRX-Li_x_TiOF_2_ samples was investigated by field emission scanning electron microscopy (FESEM, JSM-7800F, JEOL, Japan), transmission electron microscopy (TEM, JEM-F200, JEOL, Japan) and spherical aberration-corrected transmission electron microscope (AC-TEM, JEM-ARM300F2). Powder X-ray diffraction (XRD, DMAX-2500PC, Japan) using Cu-Kα radiation and ex situ grazing-incidence synchrotron XRD (BL14B, SSRF, Shanghai) was performed on as-prepared samples to gain the structure and composition. In situ XRD measurement was taken on multifunctional X-ray diffractometer (XRD, Smartlab 9KW, Rigaku, Japan) for the first cycle at 2θ values ranging from 10° to 90° with a scanning rate of 10° min^−1^, in which Be foil was employed as current collector. The stoichiometry of the as-synthesized DRX-Li_x_TiOF_2_ was determined by inductively coupled plasma optical emission spectroscopy (EA, Vario EL cube, Germany). The surface chemical species of the samples was studied by X-ray photoelectron spectroscopy (XPS, AXIS Supra, UK). An ASAP 2020 volumetric adsorption analyzer (Micromeritics, USA) at 77 K was used to achieve the pore structure data of the samples. The chemical valence states, electronic structures and local coordination environments of the samples were characterized by X-ray absorption spectroscopy (BL14W, SSRF, Shanghai). For detailed testing procedures and methodologies, please see the supplementary information.

### Cell Assembly and Test

For the fabrication of LICs, the anode is pre-lithiated, which the anode is charged and discharged multiple times and finally discharged to 0.1 V at a current density of 0.1 A g^−1^ in a half-cell, and then, the anode electrode is dismantled in the glove box. LICs were assembled with the pre-lithiated anode and the PDPC cathode. Anode and cathode active materials mass ratio is 1:1. Celgard 2400 and glass fiber membranes were used as separators. The electrode solution was 1 M LiPF_6_ in EC:DMC:EMC (1:1:1 by volume, 100 uL). All operations were performed in an Ar-filled glove box (H_2_O and O_2_ contents < 0.1 ppm). The temperature was controlled at 30 ± 1 °C throughout the entire testing process.

Cyclic voltammetry (CV) test of the half-cells recorded by IviumStat (Ivium Technologies BV, The Netherlands and Biologic SP200, France). Electrical impedance spectroscopy (EIS) test was recorded by a CHI760E (Shanghai, China). EIS was carried out with a frequency range from 0.01 to 100,000 Hz. Galvanostatic charge/discharge measurements and life span tests for half-cell and LICs used a battery test system (Land CT2001A model, Wuhan Land Electronics, Ltd.). The voltage monitoring on individual electrodes through a three-electrode cell was carried out (Biologic SP200, France). The energy density (E, Wh kg^−1^) of LICs can be evaluated by the constant discharge current (I), the cell voltage (V) and the start and end of discharge time (t_1_ and t_2_) according to the following equation:

1 $$\mathrm{E}={\int}_{\mathrm{t}1}^{\mathrm{t}2}\mathrm{IVdt}$$

The power density (P, W kg^−1^) of LICs can be achieved by the energy density (E) and the discharging time (t) according to the following equation:

2 $$\mathrm{P}=\mathrm{E}/\mathrm{t}$$

### Computational Methods

This study integrated a multiscale computational approach combining cluster expansion models, Monte Carlo simulations and first-principles calculations. Specifically, a cluster expansion Hamiltonian for the Li–Ti–O–F system was first constructed by fitting a large set of first-principles data to enable efficient prediction of energies across different atomic arrangements. Based on this, Monte Carlo simulations were performed to systematically search for and identify the stable cation/anion-disordered structures of the material near room temperature, while also statistically analyzing the lithium-ion migration network within them. Finally, using density functional theory, accurate calculations of energy, voltage and lithium-ion migration barriers were carried out on the selected key structures, thereby revealing the structure–property relationship and the mechanism behind the high-rate performance of this disordered rock-salt material at the atomic scale. For detailed computational procedures and methodologies, please see the supplementary information.

## Results and Discussion

**Fig. 1:**
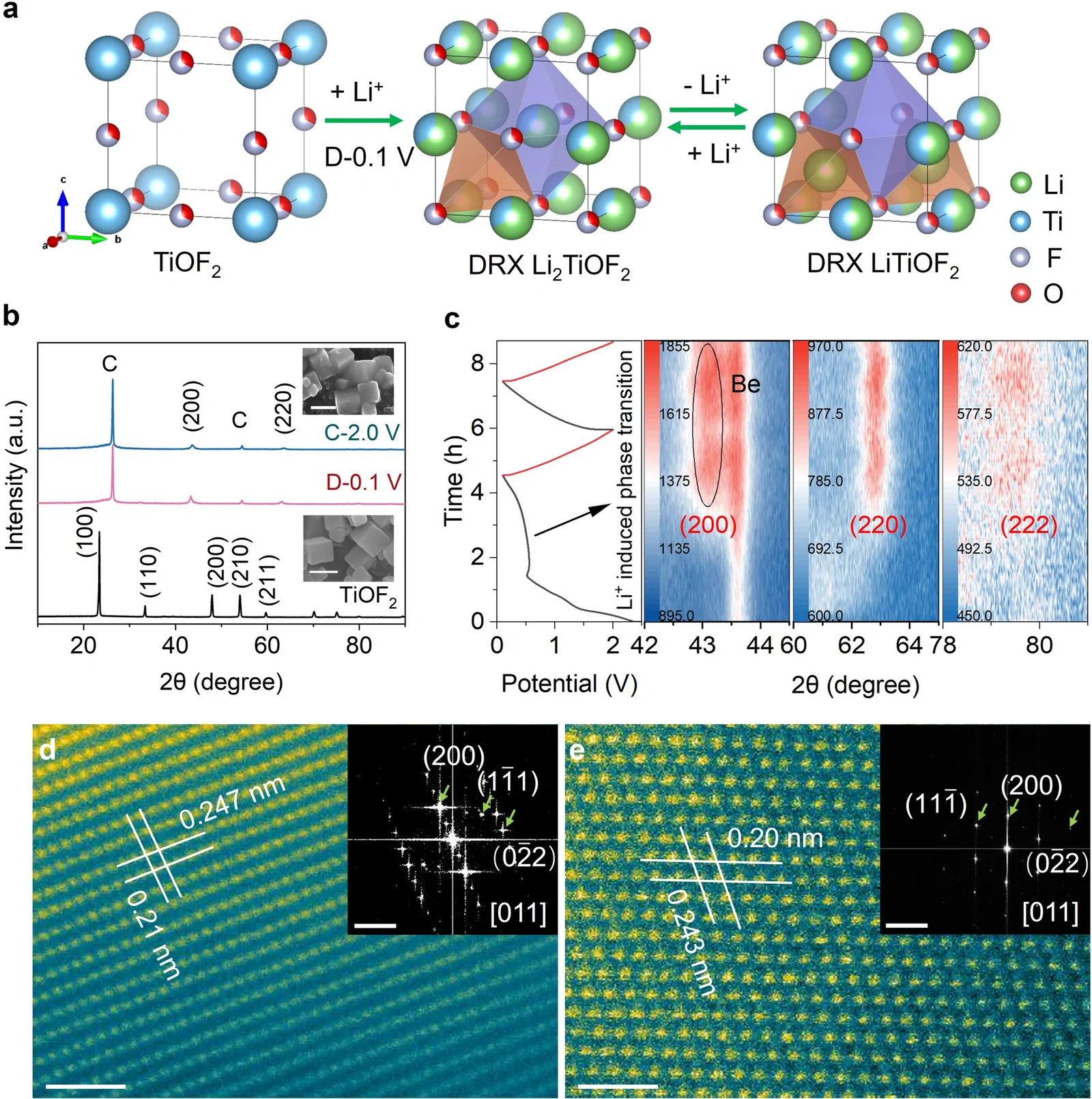
**a** Schematic of the lithium-ion storage mechanism: the electrochemically driven TiOF_2_ to DRX-Li_x_TiOF_2_ transformation and maintain DRX structure during cycling with Li^+^. The red balls represent O, gray balls represent F, blue balls represent Ti, green balls represent Li, the orange tetrahedron represents Li in tetrahedral sites, and the purple octahedron represents the Li/Ti shared octahedral sites. **b** XRD patterns of TiOF_2_, D-0.1 V and C-2.0 V. Inset is SEM image of TiOF_2_ powder and D-0.1 V (scale bar: 0.5 μm). **c** In situ XRD patterns and corresponding initial lithiation and delithiation curves at 0.07 A g^−1^. **d** HAADF-STEM (scale bar: 1 nm) and FFT images (scale bar: 5 1/nm) of the D-0.1 V. **e** HAADF-STEM (scale bar: 1 nm) and FFT images (scale bar: 5 1/nm) of the C-2.0 V

DRX-Li_x_TiOF_2_ was prepared by electrochemically induced cubic to rock-salt phase transformation of TiOF_2_ (Fig. 1a). TiOF_2_ microcubes (0.2–0.5 μm in size, Fig. 1b) were synthesized via a solvothermal method. Following the first lithiation, the as-prepared TiOF_2_ is observed to transition from an initial cubic structure (Pm-3m) to a DRX structure, the latter characterized by the Fm-3m space group (Fig. 1a). A two-stage discharge profile is also observed: an initial linear slope followed by a plateau near 0.7 V (vs. Li^+^/Li); this plateau is indicative of the phase transformation from a cubic to a DRX structure (Fig. S1).

The pristine XRD peaks for cubic TiOF_2_ basically disappear, while two new peaks emerge around 43° and 62° arising from XRD pattern after initial lithiation (Fig. 1b). These two peaks can be assigned to (200) and (220) reflections of DRX-Li_x_TiOF_2_. In situ XRD analysis further revealed a consistent variation trend (Fig. 1c). Upon emergence at the ~ 0.7 V plateau (vs. Li^+^/Li), the characteristic rock-salt (200) and (220) peaks remain stable throughout all subsequent lithiation and delithiation cycles. It has been observed that the (200) and (220) peaks shift slight to lower angles during Li^+^ insertion into the electrode; however, they will shift back during the subsequent delithiation process. This reversible peak shift was consistently observed in the second and later cycles [22, 23]. The varied law of XRD (ex and in situ) patterns confirm that irreversibly structural transformation from TiOF_2_ to DRX-Li_x_TiOF_2_ and the electrochemical behavior of DRX-Li_x_TiOF_2_ is reversible without phase change. A clear correlation is observed between the pronounced structural evolution and the distinct voltage profile of the initial cycle, the latter of which differs significantly from subsequent cycles, which suggests that newly formed DRX-Li_x_TiOF_2_ is reversibly cycled and remained unchanged even after operating 10 cycles and more than 1000 cycles (Fig. S2). Similar phase transformation trend was observed in ex situ grazing-incidence synchrotron X-ray diffraction (Fig. S3). The major peaks are indexed to a Li_x_TiOF_2_ cubic rock-salt phase. Due to the high sensitivity and resolution of synchrotron XRD, some diffraction peaks of untransformed pristine TiOF_2_ are detected [24]. We simulated ex situ grazing-incidence synchrotron XRD patterns using the DRX structure from MC calculations and observed close agreement with the experimental data.

The unit cell volume of DRX-Li_x_TiOF_2_ (1 < x < 2) undergoes a minimal expansion of only 2.8% after full lithiation, as measured by the shift in the (200) XRD peak (Fig. S4). As expected, the small volume change in the DRX-Li_x_TiOF_2_ (1 < x < 2) unit cell gives rise to the limited thickness variation (change rate < 10%) of electrode during the lithiation and delithiation, as confirmed by cross-sectional SEM images (Fig. S5). The reversible electrochemical behaviors coupled with no phase change suggest that the newly formed DRX-Li_x_TiOF_2_ exhibits exceptional stability as a host framework for intercalation. The Li/Ti ratios of DRX-Li_x_TiOF_2_ were measured by ICP and XPS. ICP results (Table S1) indicate a Li/Ti molar ratio of 2.95 upon discharging to 0.1 V, a value which incorporates the lithium within the solid–electrolyte interphase (SEI). The decrease in the Li/Ti molar ratio to 1.89 at 2.0 V demonstrates a reversible cycling capability of 1.06 Li per formula unit. The deep etch XPS test (Fig. S6) was performed on a sample upon lithiated and delithiated stages. Following 1000 s of etching, the Li/Ti molar ratios stabilized at 2.28 (discharged to 0.1 V) and 1.19 (charged to 2.0 V). The presence of a SEI layer within the material results in a lightly increased Li/Ti ratio following deep etching, with a ratio greater than 2 being observed. According to the XPS results, there was 1.09 lithium undergoing percolation, which is highly consistent with the ICP analyses. The ICP, XPS and electrochemical analysis charge/discharge results confirm that phase transition between DRX-Li_2_TiOF_2_ and DRX-Li_1_TiOF_2_ is completely reversible.

The structure evolution of cubic TiOF_2_ to a DRX phase was further elucidated using TEM and AC-TEM. Figure S7a shows that the cube-shaped TiOF_2_ is well crystallized. The lattice spacings of 0.37 and 0.26 nm, measured from the HAADF–STEM image (Fig. S7b), correspond to the (100) and (110) planes of the cubic phase, respectively. This cubic structure is further confirmed by the fast Fourier transform (FFT) pattern (Fig. S7c), in which the spots can be indexed to the (100) and (110) crystallographic planes. EDS characterizations confirm a homogeneous distribution of Ti, O and F atoms (Fig. S7d–g). After the lithiation down to 0.1 V, the DRX-Li_x_TiOF_2_ maintain the cubic morphology and well crystallized (Fig. S8). Figure 1e shows HAADF-STEM image along the [011] zone axis of D-0.1 V. The corresponding FFT result exhibits the (1$\stackrel{\mathrm{-}}{1}$1), (200) and (0$\stackrel{\mathrm{-}}{2}$2) planes, confirming the formation of cubic rock-salt structure. Electrochemically induced method tends to form the rock-salt phase [25, 26]. In addition, the presence of a thin SEI (∼7 nm) on the electrode surface, as shown in Fig. S9, is corroborated by the corresponding SEM observation in Fig. S5. DRX-Li_x_TiOF_2_ maintains a rock-salt structure after the delithiation up to 2.0 V, which is confirmed by HAADF-STEM, HRTEM and SAED (Figs. 1f and S10). SEM images further show that the formed DRX-Li_x_TiOF_2_ in the electrode preserves the cubic morphology of TiOF_2_ powder during the initial lithiation and delithiation process and subsequent long-term cycles (Figs. S11 and S12). The structural evolution results are apparent different from previous studies in which cubic TiOF_2_ would be transformed into a mixture of “Li_z_TiO” + LiF after the lithiation [27–29]. The evolution of cubic TiOF_2_ to a DRX structure is more like electrochemically induced phase transformation from V_2_O_5_ to DRX Li_3+x_V_2_O_5_ [25, 30, 31].

**Fig. 2:**
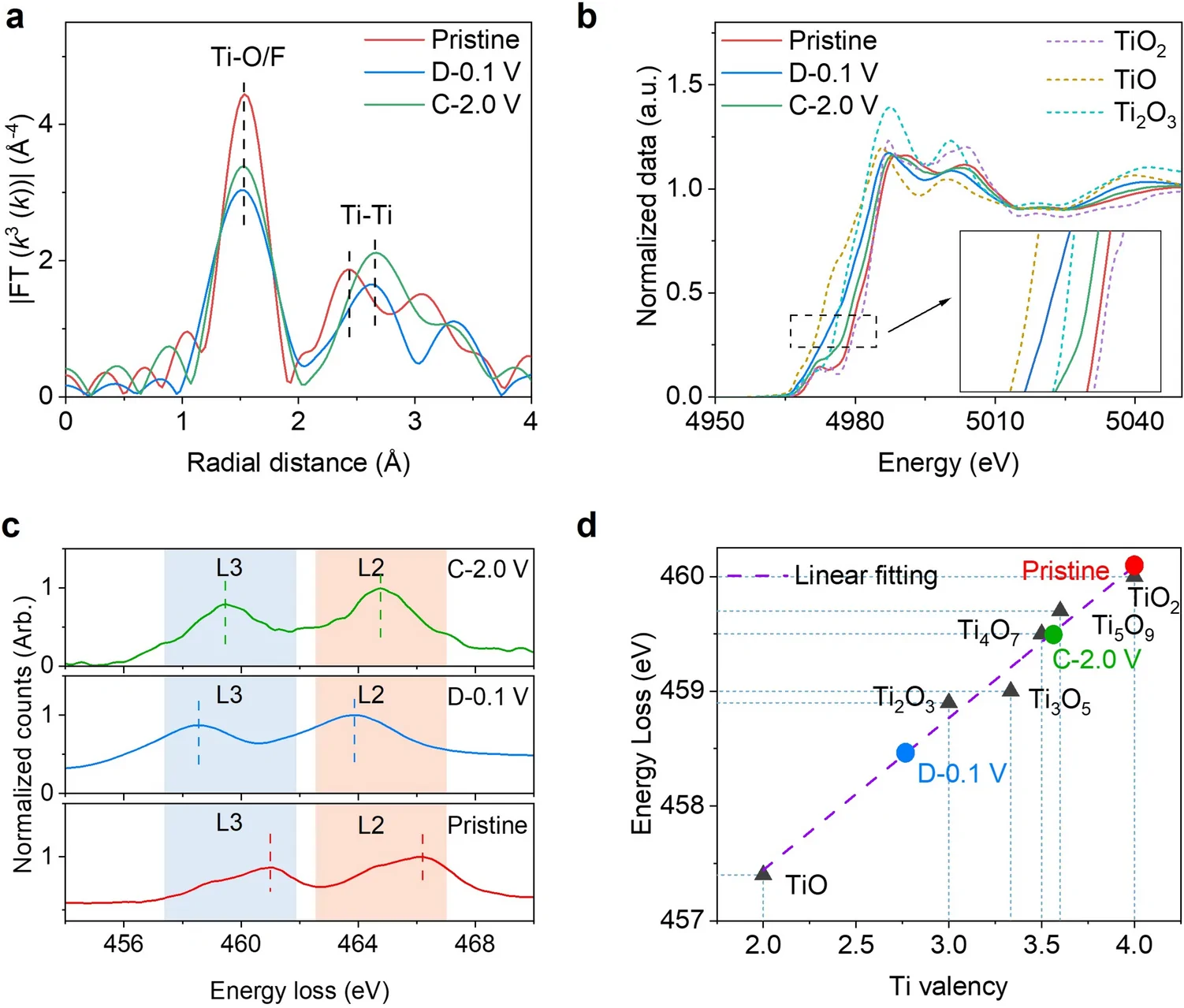
**a** *k*^3^-weighted Fourier transform magnitudes of Ti K-edge EXAFS spectra at different voltages during the first cycle. **b** Normalized ex situ Ti K-edge XANES spectra obtained at different voltages during the first cycle. **c** EELS spectrum of Ti. **d** Energy position determined from EELS of several Ti reference compounds as indicated as a function of the Ti valency

Ex situ extended X-ray absorption fine structure (EXAFS) spectroscopy was used to probe the local structural evolution of Ti during the initial lithiation/delithiation cycle. EXAFS spectra for Ti, TiO, Ti_2_O_3_ and TiO_2_ standards are provided in Fig. S13. A strong peak centered in 1.54 Å is assigned to the Ti–O and/or Ti–F bonds, and two prominent peaks centered at 2.43 and 3.06 Å are assigned to the Ti–Ti bonds for the pristine sample (Fig. 2a) [32]. Upon the discharging to 0.1 V, Ti–Ti peaks shift to longer bond distances (∆_shift_ =  ~ 0.2–0.3 Å) as a consequence of the structural evolution resulting in the formation of a DRX phase (Fm-3 m) from an initial cubic structure (Pm-3 m). Furthermore, the reduction in the Ti–O/F and Ti–Ti peak intensities after lithiation is due to the disordered occupation of octahedral sites by Li and Ti in the DRX structure, which disrupts the Ti periodicity and weakens the Ti–Ti interaction through Li shielding [33]. Upon the charging to 2.0 V, no apparent peak shift is observed for either Ti–O/F peaks or Ti–Ti peaks, suggesting the stable rock-salt structure without the phase transformation during the delithiation. The increased intensity of the Ti–O/F peaks and Ti–Ti peaks is probably due to the reduced Li atoms occupied in octahedral sites and the reduced shielding effect between Li and Ti–Ti interaction. To monitor the Ti valence state evolution during Li^+^ insertion and desertion, ex situ Ti K-edge XANES measurements were taken (Fig. 2b). The XANES spectrum obtained from the pristine sample exhibited close similarity to that of TiO_2_ (Ti^4+^ reference) [34], suggesting a predominant Ti^4+^ state in bulk. Upon discharging to 0.1 V (versus Li^+^/Li), the Ti K-edge position shifted toward an energy between Ti_2_O_3_ (Ti^3+^ reference) and TiO (Ti^2+^ reference), which indicates that the materials predominantly consist of Ti^3+^ and Ti^2+^ [34, 35]. After the subsequent charging to 2.0 V, the Ti oxidation state is restored to a level between + 3 and + 4. This phenomenon arises from the fact that Li^+^ ions do not fully detach from the electrode, which is confirmed by Li/Ti ratios of electrode determined via the XPS (Fig. S7) and ICP (Table S1).

To track the chemical state evolution, ex situ Ti 2*p* core level XPS spectra were obtained at various lithiation and delithiation stages (Fig. S14). In the pristine electrode's Ti 2*p* spectrum, the two primary peaks at 465.5 and 459.7 eV signify Ti^4+^ [36]. Discharging to 0.1 V (vs. Li^+^/Li) shifts these peaks to lower binding energies, revealing a decrease in the Ti valence state—a finding corroborated by the XANES spectra, which show reduction to Ti^3+^/Ti^2+^. The Ti 2*p* spectra could be fitted with four peaks at 455.1, 458.4, 461.3 and 464.25 eV, which orrespond to Ti^3+^ and Ti^2+^ species [37, 38]. The Ti 2*p* peak shifts slightly back to higher binding energy upon recharging to 2.0 V, accompanied by a reduction in the Ti^3+^ fraction and a rise in the Ti^4+^ fraction. The Ti L_3,2_-edge EELS spectra further confirm the reduction of the Ti valence state, as evidenced by a shift of the edge onset to lower energies after lithiation [39]. Upon charging to 2.0 V, Ti L_3,2_ EELS spectra shift to higher energy with increasing oxidation state (Fig. 2c). Figure 2d shows the correspondence between the valence and energy of Ti, from which it follows that 0.9 electrons can be reversibly deembedded during charging and discharging. EELS measures the average valence of all Ti atoms in the detection volume, and this spatial averaging can lead to a slight underestimation of the net electron transfer compared to stoichiometry-based methods like ICP or XPS, which directly quantify the total charge-compensating Li^+^. The above characterizations demonstrate that the structural transformation from cubic TiOF_2_ to DRX-Li_x_TiOF_2_ undergoes multielectron redox in Ti (> 1:1 Li/Ti) upon lithiation, whereas the newly formed DRX-Li_x_TiOF_2_ exhibits the electrochemical activity through the reversible Ti^2+^/Ti^4+^ redox reactions, achieving a transfer of 1.08 electrons during charge–discharge cycles and in accordance with the ICP and XPS test analysis.

**Fig. 3:**
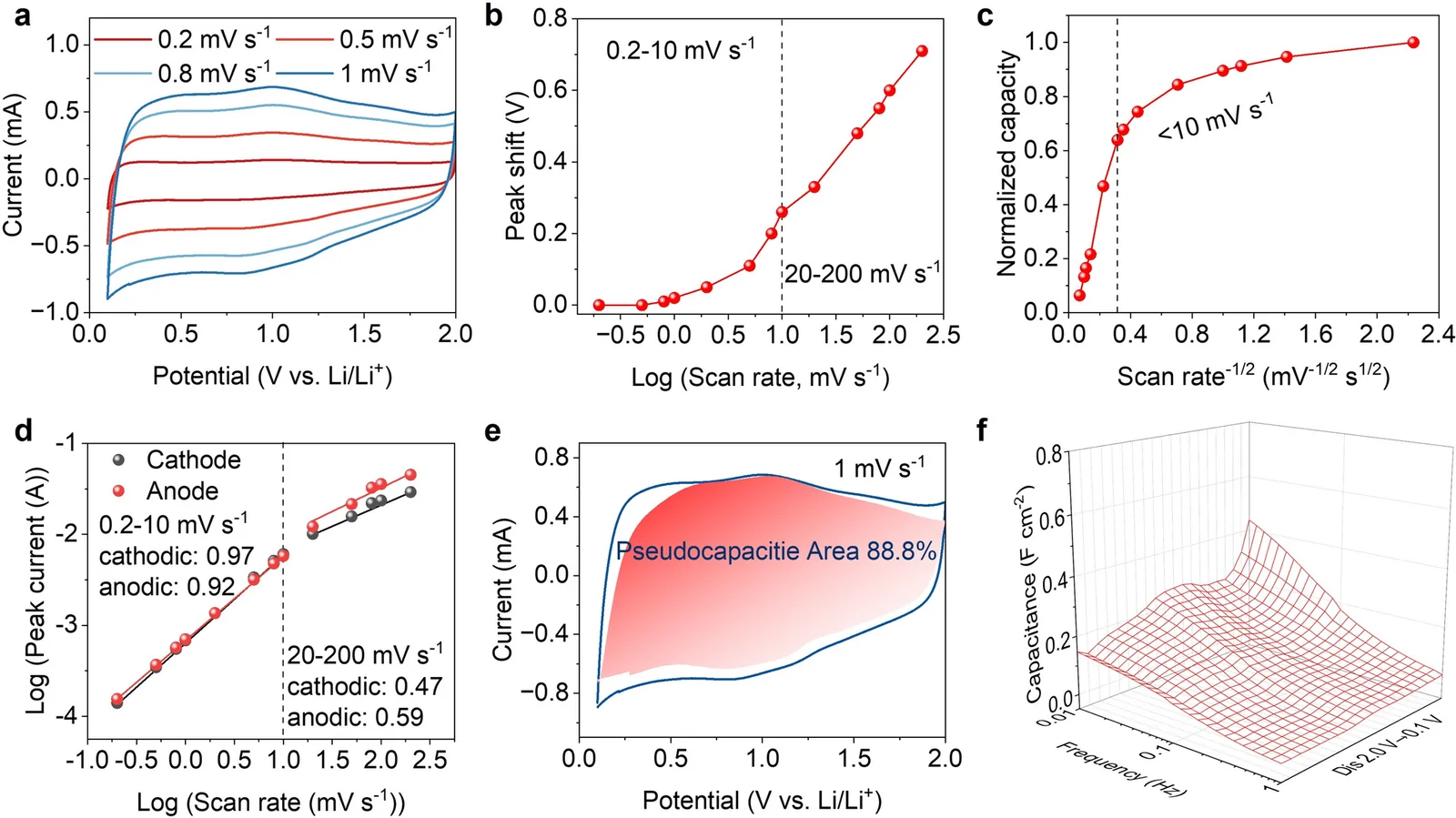
Electrochemical performance of DRX-Li_x_TiOF_2_. **a** CV profile at the different scan rates. **b** Variation of the peak voltage with the sweep rate. **c** Capacity versus *v*^−1/2^ allows for the separation of diffusion-controlled capacity from capacitive-controlled capacity. **d** *b*-values determination of the anodic for different active material mass. **e** Voltametric response for DRX-Li_x_TiOF_2_ electrode at a sweep rate of 1.0 mV s^−1^. The capacitive contribution to the total current is shown in the red region. **f** 3D Bode plots showing the normalized capacitance (*C*) versus frequency and potential for capacitive

A half-cell consisting of a DRX-Li_x_TiOF_2_ working electrode and a Li–metal counter/reference electrode was assembled to evaluate its electrochemical Li^+^ storage behavior. The cyclic voltammograms (CVs) of DRX-Li_x_TiOF_2_ electrode exhibit a typical quasi-rectangular shape with scan rates from 0.2 to 10 mV s^−1^, suggesting a pseudocapacitive behavior (Figs. 3a and S15). The CV curves reveal only minor redox features around 1.0 V, which exhibit a subtle peak shift (ΔE <  ~ 0.3 V) at sweep rates below 10 mV s^−1^ (Fig. 3b). Over 20 mV s^−1^ (Fig. S15), the quasi-rectangular CV become distorted, primarily due to reaching diffusion-limited behavior and increasing the ohmic contribution [40]. This quasi-rectangular CVs of DRX-Li_x_TiOF_2_ is very similar to CVs of redox pseudocapacitive materials without apparent redox peaks, such as RuO_2_ [14], MnO_2_ [15] and nitrogen-doped mesoporous carbon [41], but differ from CVs of intercalation pseudocapacitive materials with apparent redox peaks, such as Nb_2_O_5_ [18], TiO_2_(B) [19], H_2_Ti_2_O_7_ [42], MoO_x_ [43], nano-MoS_2_ [44]. The quasi-rectangular CV shape persists even at a high mass loading of 3.9 mg cm^−2^ (Fig. S16), demonstrating its applicability beyond thin-film electrodes. The relationship between normalized capacity and *v*^−1/2^ (0.2–200 mV s^−1^) is shown in Fig. 3c. Within Region 1 (*v* < 10 mV s^−1^), the near-constant capacity signifies a capacitive-dominated charge storage mechanism, where solid-state diffusion does not limit the kinetics. The diffusion-controlled charge storage mechanism in region 2 (20–200 mV s^−1^) manifests as a linear decrease in capacity with v^−1/2^, analogous to the behavior observed in most traditional battery electrodes. The charge storage kinetics were quantified by analyzing the log–log plot of peak current versus scan rate (Fig. 3d) according to the power law (*i* = *av*^b^) [40]. In this equation, a *b*-value of 0.5 signifies diffusion-limited (battery-type) behavior, and 1.0 denotes a capacitive process [18]. The experimental *b*-value of 0.97, which approaches unity across scan rates of 0.2–10 mV s^−1^, confirms that surface-controlled processes are dominant. The *b*-value can be improved to 0.98 (Fig. S17) for a thinner DRX-Li_x_TiOF_2_ electrode (0.25 mg cm^−2^). When the scan rate increases beyond 20 mV s^−1^, the *b*-value converges on 0.47, identifying solid-state diffusion as the dominant rate-limiting process (Fig. 3d) [43]. This diffusion limitation becomes more pronounced at higher mass loadings (Fig. S16). This observation is in agreement with other intercalative host materials with pseudocapacitive behavior [18, 31, 45]. Quantification of the capacitive contributions was carried out following the procedure of Dunn et al. [19, 46]. The charge storage mechanism is overwhelmingly capacitive, contributing 88.8% to the total current at 1 mV s^−1^ across the full voltage range (Fig. 3e). Notably, electrodes with a high mass loading of 3.9 mg cm^−2^ maintain a significant capacitive percentage of 72.8% at the same rate (Fig. S16). 3D Bode confirms that the capacitance remains constant across different potentials, confirming the pseudocapacitive features of DRX-Li_x_TiOF_2_ (Fig. 3f).

**Fig. 4:**
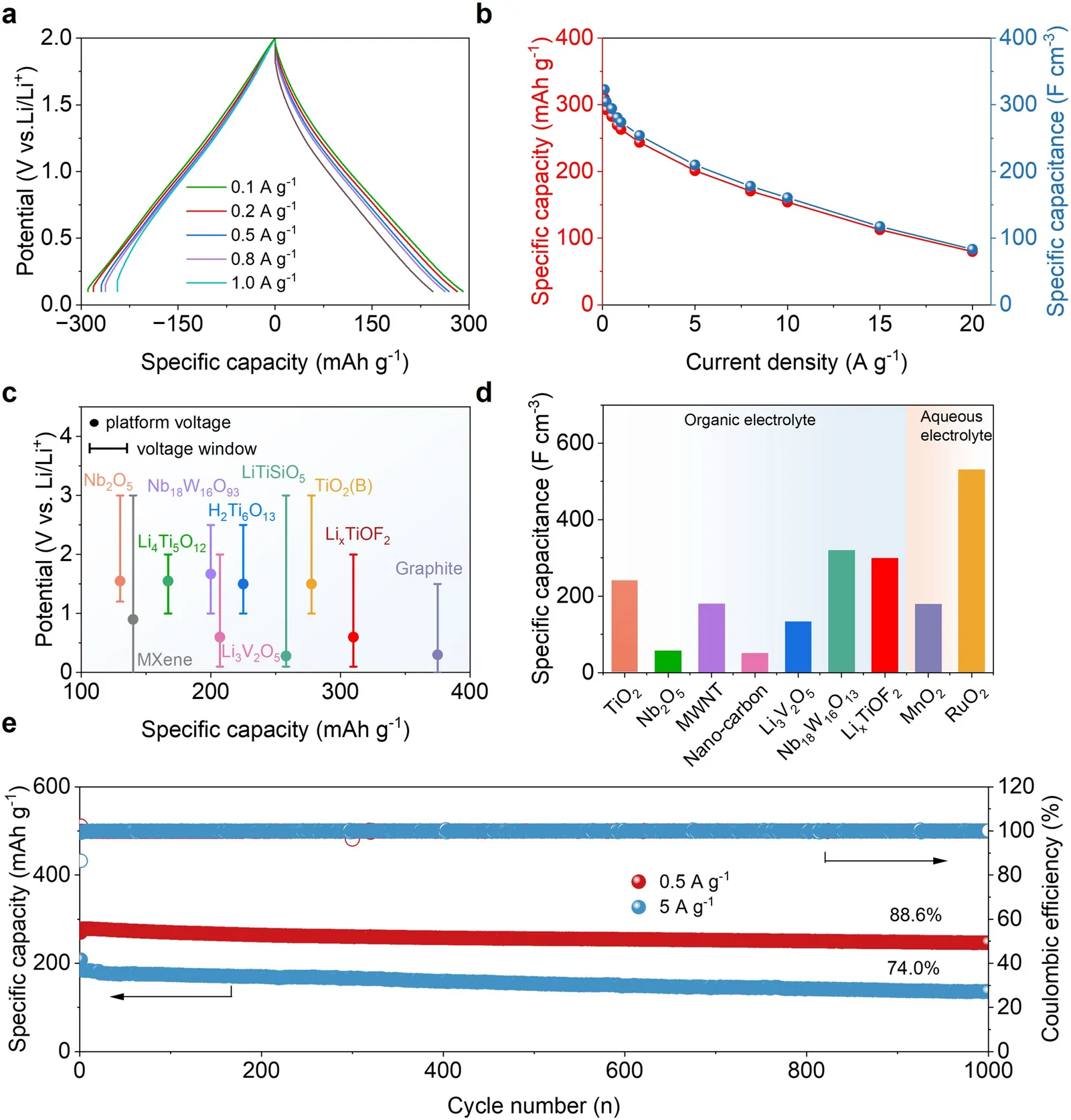
**a** Galvanostatic charge/discharge profiles at various current densities. **b** Rate capability from 0.1 to 20 A g^−1^ for DRX-Li_x_TiOF_2_. **c** Specific capacity value and working potential range of DRX-Li_x_TiOF_2_ anodes and comparison with other commonly used anode materials. **d** Volumetric capacitance of DRX-Li_x_TiOF_2_ anodes and comparison with other commonly used anode materials. **e** Long-term cycling stability for DRX-Li_x_TiOF_2_ anodes at 0.5 and 5.0 A g^−1^

The galvanostatic charge–discharge (GCD) curves in Fig. 4a exhibit almost triangular shape with the minimal hysteresis, consistent with the characteristic of capacitor-like materials (e.g., activated carbon), providing further evidence for the pseudocapacitive nature of the charge storage. Although such behavior has rarely reported for the intercalation pseudocapacitive materials [18, 19] in organic electrodes, similar behavior has also been reported for other surface redox pseudocapacitive materials, such as 1 T-MoS_2_ [47], MXene [48], RuO_2_ [14] and MnO_2_ [15] in aqueous electrolytes. Figure 4b shows that the electrode delivers an extremely high capacitance of 587 F g^−1^ (310 mAh g^−1^) at 0.1 A g^−1^. The electrode exhibits outstanding rate capability (Fig. 4b), delivering an initial capacitance of 587 F g^−1^ (310 mAh g^−1^) at 0.1 A g^−1^. This value attenuates to 291 F g^−1^ (154 mAh g^−1^) at a high current density of 10 A g^−1^, equating to a 49.7% retention. Impressively, a significant capacity of 176 F g^−1^ (93 mAh g^−1^) is preserved even at 20 A g^−1^. The Li^+^ storage capability of DRX-Li_x_TiOF_2_ significantly surpasses that of typical battery-type materials [49, 50] and is competitive with state-of-the-art pseudocapacitive electrodes (Fig. 4c) [7, 18, 31, 42, 48, 51, 52]. Additionally, based on the electrode density of 0.51 g cm^−3^ for DRX-Li_x_TiOF_2_, the calculated volumetric capacitances reach 299.5, 252.9, 148.4 and 89.0 F cm^−3^ at current densities of 0.1, 1.0, 10 and 20 A g^−1^, respectively, which is higher than that of MWNT [53], nanostructured carbon [54], Nb_2_O_5_ [18], TiO_2_(B) [55] in organic electrolytes as well as RuO_2_ [56], MnO_2_ [57], Li_3_V_2_O_5_ [31] electrodes (Fig. 4d). The DRX-Li_x_TiOF_2_ electrode presents a good cycling stability. The LixTiOF_2_ electrode maintained near-100% coulombic efficiency over 1,000 cycles (Fig. 4f), while demonstrating substantial capacity retentions of 88.6% at 0.5 A g^−1^ and 74.0% at 5.0 A g^−1^. We have supplemented the cycling performance test at a low current density of 0.1 A g^−1^. The results show that DRX-Li_x_TiOF_2_ delivers an initial reversible capacity of approximately 320 mAh g^−1^ at 0.1 A g^−1^ (Fig. S18). Impedance spectra were analyzed at different voltages and after various numbers of cycles (Fig. S19). The results show that after 10 cycles, DRX-Li_x_TiOF_2_ exhibits a lower charge transfer resistance (R_ct_) than the pristine sample, indicating enhanced interfacial charge transfer and more efficient lithium-ion diffusion within the DRX structure. During the first charge–discharge process, the impedance remains low across all measured voltages, further reflecting the rapid lithium-ion migration enabled by this network. Based on GITT measurements, the lithium-ion diffusion coefficient ($\hbox{D}_{{\rm Li}^{+}}$) of the material was calculated to be in the range of 1.20 × 10^−9^ to 7.08 × 10^−9^ cm^2^ s^−1^ during lithiation/delithiation (Fig. S19). These high values directly confirm the fast lithium-ion transport kinetics in DRX-Li_x_TiOF_2_, providing experimental support for its excellent high-rate performance.

**Fig. 5:**
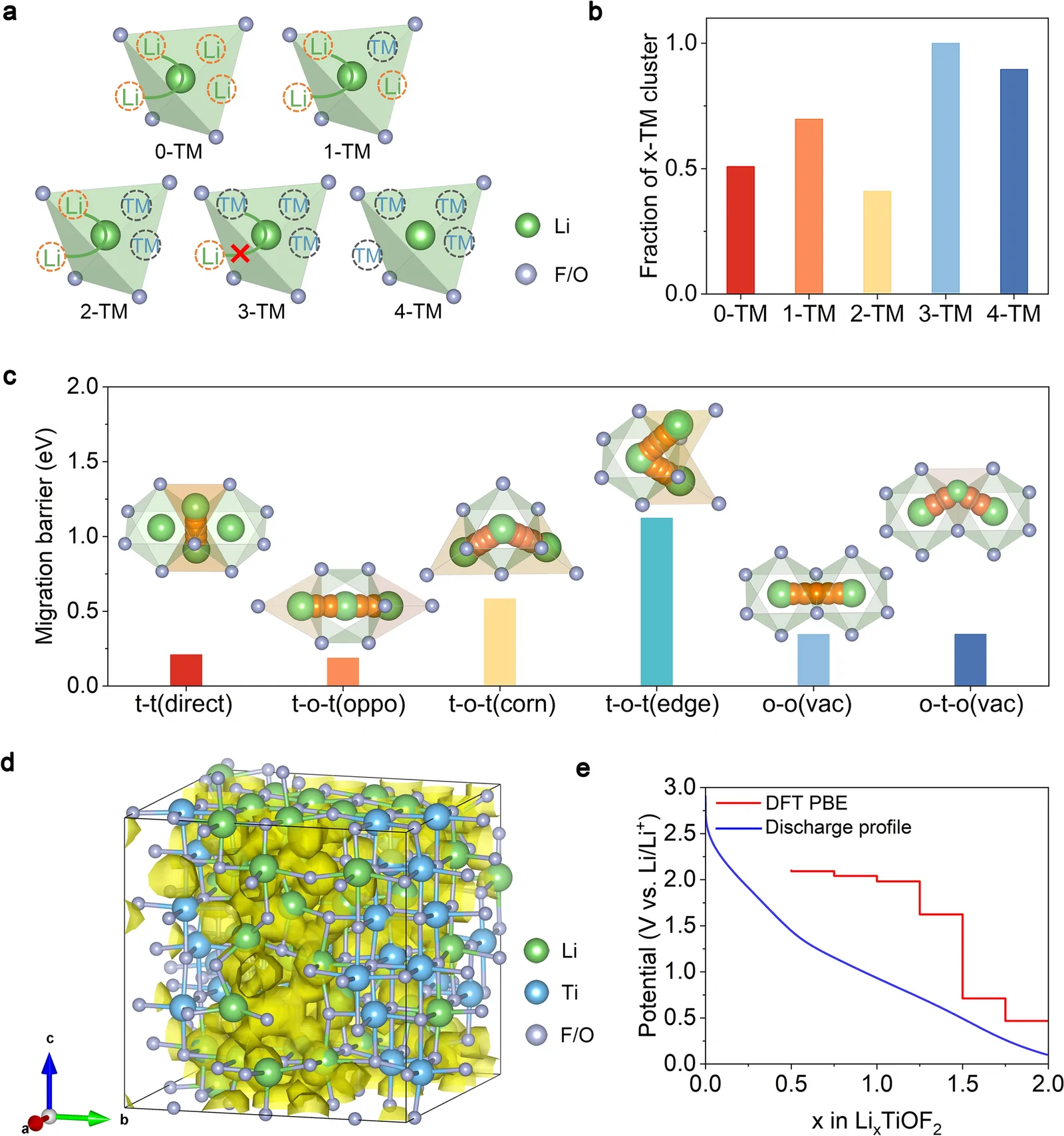
**a, b** Occurrence of various tetrahedral clusters (0-TM, 1-TM, 2-TM, 3-TM and 4-TM) in cation-disordered rock-salt Li_2_TiOF_2_ as compared to the random limit. **c** Li migration barriers in Li_2_TiOF_2_. Calculated NEB barriers for possible Li migration hops in Li_2_TiOF_2_. **d** Li^+^ probability density of Li_1.5_TiOF_2_ at 1000 K from AIMD simulations. **e** Comparison between experimental and computed voltage profiles of DRX-Li_x_TiOF_2_ (0 < x < 2) upon electrochemical cycling

The rapid Li^+^ diffusion mechanism in DRX-Li_x_TiOF_2_ was explored using a combined approach of density functional theory (DFT)-based cluster expansion (CE) and MC simulations. The initial structural model was based on a DRX-Li_2_TiOF_2_ framework, characterized by full occupancy of the 4*a* anionic sites by O^2−^/F^−^ ions, while the octahedral 4*b* sites were randomly occupied by Li^+^ and Ti cations. The atomic-scale equilibrium short-range ordering, as determined by MC simulation, allows for a detailed analysis of the lithium transport environment from the local to the macroscopic level. The local environment in DRX materials, governed by various cation clusters, directly influences Li^+^ transport, where tetrahedral clusters play the most critical role as conduction pathways [58]. The statistical distribution of different tetrahedral clusters is shown in Fig. 5a. The Li^+^ transport efficiency is largely dictated by a percolating network of 0-TM channels, owing to their minimal migration energy barriers. Based on the MC model and a standardized statistical analysis of Li^+^ transport pathways, 0-TM channels represent 51% of all tetrahedral clusters in DRX-Li_x_TiOF_2_, which underscore their role as the primary channels for Li migration. These channels are thus critical for achieving high-rate performance. In contrast, pathways involving transition metal (TM) ions (e.g., 1-TM or 2-TM) or high-TM-coordination environments (e.g., 3-TM or 4-TM) significantly increase energy barriers or create kinetic traps, hindering Li migration [59–61]. To quantifying the effect of replacing O with F, statistical coordination analyses were carried out by using MC simulations (Fig. S20). Figure S20a reveals F anions exhibit exclusive preference for high Li coordination, dominated by FLi_6_ configurations with decreasing proportion through FLi_5_ to FLi_2_, while FLi_1_ and FLi_0_ coordination is absent, confirming F avoids direct bonding with Ti. Conversely, Fig. S20b demonstrates O atoms favor Ti coordination, showing predominant OLi_1_ occupancy while lacking OLi_6_/OLi_5_. This complementary coordination contrast originates from higher electronegativity of F, driving Li toward F. Correspondingly, Fig. S20c visually manifests this chemical segregation, which Li-saturated domains (green) cluster around F (purple), while Ti (blue) concentrates in O-rich zones (red). Such phase separation establishes continuous Li-enriched highways devoid of transition metals, enabling barrier-free 0-TM diffusion channels. Therefore, we assume that the composition of 0-TM after fluorination is mainly LiF_4_ clusters [59, 62, 63]. MC results at 300 K further confirm that these 0-TM Li diffusion channels interconnect to form a 3D framework, which are highlighted in green in Fig. S21. As evidenced by the 0-TM networks, the Li migration channels exhibit excellent connectivity, forming an extensive skeleton of pathways.

A deeper understanding of the Li migration mechanisms in DRX-Li_2_TiOF_2_ was achieved by calculating the kinetically resolved activation barriers for different Li migration pathways in representative SQoS structure (detailed in the Computational Methods section), taking into account the possible influence of the local environment. In conventional DRX cathodes, the primary Li^+^ diffusion proceeds through an octahedral–tetrahedral–octahedral (o–t–o) pathway, where Li hops between octahedral sites via tetrahedral intermediates [64]. For a DRX-Li_2_TiOF_2_ anode with abundant tetrahedral vacancies, CI-NEB-DFT results elucidate the primary Li^+^ migration pathways in subsequent lithiation (Fig. 5c). The most favorable mechanisms are the direct tetrahedral–tetrahedral (t–t) and the opposing tetrahedral–octahedral–tetrahedral (t–o–t) migrations. The direct t–t pathway involves a Li jump into an adjacent tetrahedral site with calculated migration barrier of 0.272 eV (Fig. S22). The opposing t–o–t mechanism, involving cooperative Li hops from one 0-TM tetrahedral site to the opposite tetrahedral site via intermediate octahedral site where the two tetrahedral sites are located on opposite sides of this octahedron, has a comparable migration barrier of 0.273 eV (Fig. S22) [25, 26]. The situation above is the case when Li in Li_2_TiOF_2_ is fully occupied. For the DRX structure with a small number of vacancies, the o–o (vac) and the o–t–o mechanisms have relatively low barriers of 0.370 and 0.371 eV, respectively. The structural studies emphasize the value of low Li migration barriers and 3D percolation network consisting of 0-TM channel, which enable rapid Li^+^ transport [59]. To visualize the trajectory of Li^+^ transport, we explored the typical intermediate state of Li_1.5_TiOF_2_ using ab initio molecular dynamics simulations. Figure 5d illustrates the Li probability densities at 1000 K. The significant Li probability density extending between these highly occupied tetrahedral sites points to pervasive Li occupancy within the diffusion channels. The enlarged image in Fig. S23 confirms continuous 3D diffusion channels through spatially connected isosurfaces, demonstrating lithium migration, which is consistent with neb calculation results. The consistent probability density distribution across octahedral and tetrahedral sites is indicative of a flat energy profile, enabling smooth Li^+^ transport along the diffusion pathways. Overall, these results demonstrate that the interconnected 0-TM channels in DRX-Li_2_TiOF_2_ form a three-dimensional percolation network with low activation barriers, accounting for the fast Li transport [60, 61, 65, 66]. This three-dimensional percolation network directly enables the "percolating pseudocapacitance" storage mechanism—lithium ions (Li^+^) can be rapidly stored and released through the interconnected 0-TM channels that permeate the bulk material, bypassing the constraints of traditional surface reactions or confined diffusion, thereby achieving capacitor-like high-rate charge storage within the bulk phase.

To gain deeper insight into the delithiation mechanism of Li^+^ in DRX-Li_x_TiOF_2_, the Li vacancy ordering structures based on the initial SQoS supercell (the fully occupied Li_2_TiOF_2_) at different Li compositions were enumerated for obtaining the configurations with the lowest Ewald energy. DFT calculations were then performed to fully relaxed these the Li vacancy ordering structures and investigate the structural evolution during delithiation. Figure S24 shows the Li-ion site occupancies determined from the most stable DFT-relaxed structures and their dependence on the overall composition. During delithiation, DRX-Li_x_TiOF_2_ undergoes a multistep structure evolution. Typically, upon full lithiation (x = 2), from the DFT-relaxed structure of Li_2_TiOF_2_ is shown in Fig. S24. The [Li_2_Ti]_4b_[OF_2_]_4a_ configuration, with all octahedral sites fully occupied by Li and Ti ions, is in agreement with the GSAS-II refinement results (Fig. S25). With further deintercalation of Li (x = 1.5), 2.4% Li are shifted from the octahedral to the tetrahedral positions. At x = 1, 9.6% Li displaced to tetrahedral positions. In further deintercalation of Li from DRX-Li_x_TiOF_2_ (x = 0.5), Li shows a disordered distribution in tetrahedral and octahedral sites and the presence of a large number of Li tetrahedral and octahedral sites in the DRX structure. Thus, Li can be transported quickly within the DRX structure with no migration barrier when x = 1. In order to verify the above conclusions, we further explored the typical intermediate state at composition of Li_36_Ti_36_O_36_F_72_ (i.e., Li_1_TiOF_2_). Figure S26 shows the unrelaxed structure of Li_36_Ti_36_O_36_F_72_ as one Li atom is removed. It can be seen that the supercell structure has undergone a significant structural rearrangement, with a large range of Li atoms moving from octahedral sites to tetrahedral interstitial sites during relaxation. A stepwise analysis of structural optimization calculations based on this initial structure was conducted until the final fully relaxed Li_36_Ti_36_O_36_F_72_ structure was obtained, where a clearly new Li spontaneous structural rearrangement can be observed (Fig. S26). The Li atoms in the black and blue circles spontaneously generate a segment of shift from the octahedral site to the adjacent tetrahedral site during structural relaxation. The theoretical results above demonstrate that there is no migration barrier during initial lithium insertion and delithiation (Li < 2), allowing spontaneously and rapidly Li^+^ diffusion. The voltage profile for lithium deintercalation in DRX-Li_x_TiOF_2_ (0 < x < 2) was calculated. Multistep Li desertion processes are found. Agreeing well with the experimental data obtained at low current density, the PBE-computed voltage profile is presented in Fig. 5e, which provides a plausible explanation on the linear charge/discharge curves for the material. As the voltage is greater than 2.0 V, two voltage plateaus appear at 2.09 and 2.04 V for 0.5 ≤ x ≤ 1. As the voltage below 2.0 V, four voltage plateaus appear at 1.98, 1.62, 0.71 and 0.46 V for 1 ≤ x ≤ 2. In this case, ∆x = 1 mol Li^+^ remains in this material theoretically, which is close to the experimental results on the delivered specific capacity (~ 1.19 Li^+^) and the changed valence state of Ti. Besides, the PBE-calculated average voltage of 1.19 V is in reasonable agreement with the experimentally measured value (1.02 V).

**Fig. 6:**
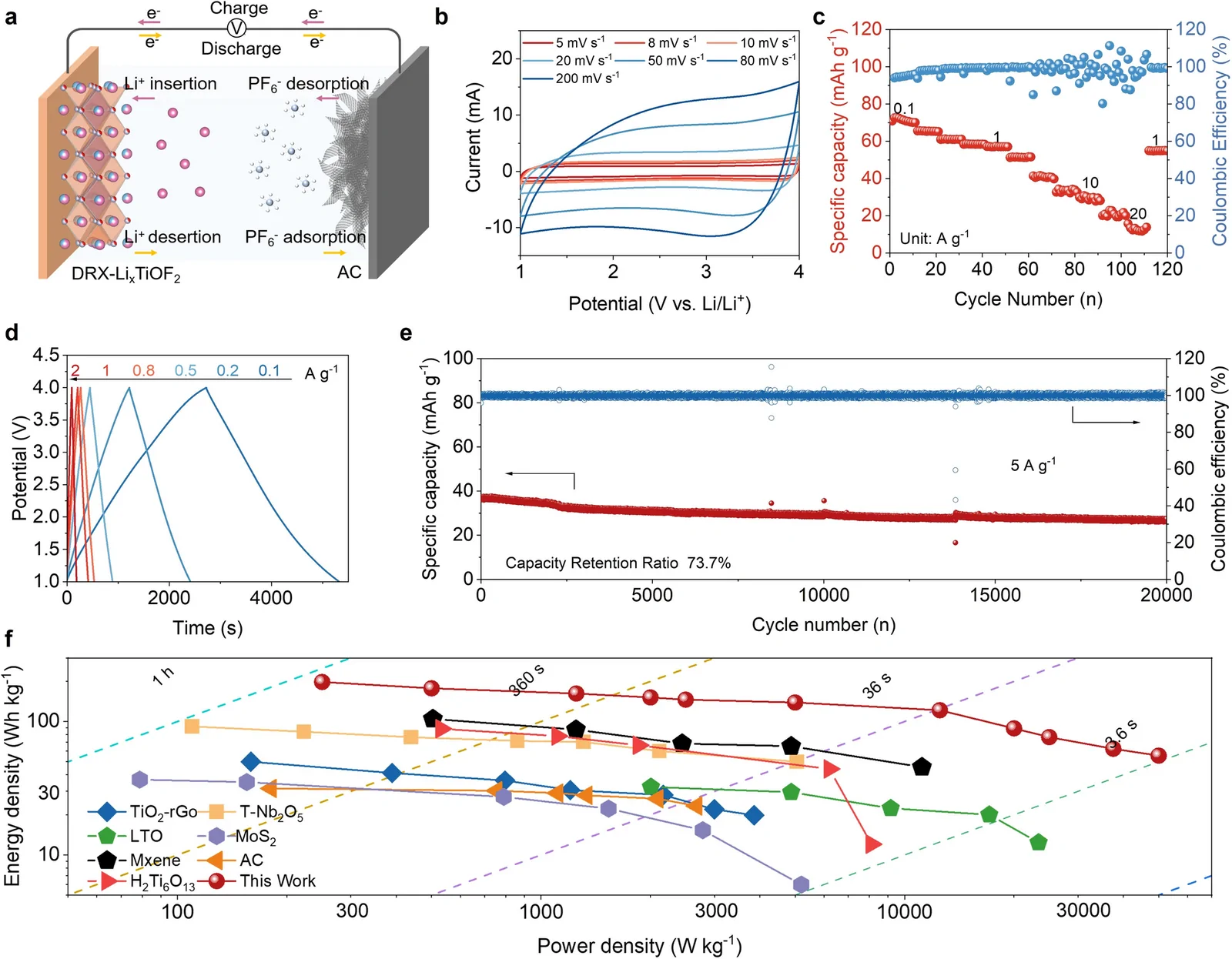
Capacity performance of LICs DRX-Li_x_TiOF_2_//AC in full-cell configuration. **a** Schematic image of the assembled LICs device. **b** CVs profile at the different scan rates for LICs DRX-Li_x_TiOF_2_//AC. **c** Rate performance under different current densities for LICs DRX-Li_x_TiOF_2_//AC. **d** Galvanostatic discharge profiles at various current densities. **e** Long-term cycling performance at 5.0 A g^−1^. **f** Ragone plots showing energy and power densities versus other reports

To evaluate the Li_x_TiOF_2_ pseudocapacitive electrode in a practical configuration, we constructed an asymmetric full cell (also known as a lithium-ion capacitor, LIC). This asymeetric device, as illustrated in Fig. 6a, incorporates a DRX-Li_x_TiOF_2_ anode and a commercial activated carbon (AC) cathode. CV was performed over a voltage range of 1.0–4.0 V at scan rates from 20 to 200 mV s^−1^ (Fig. 6b). The resulting profiles display near-rectangular shapes with minimal distortion even at 200 mV s^−1^ (discharge time ~ 20 s), indicating outstanding high-rate capability. Figure 6c further demonstrate stable operation across current densities of 0.1 to 20 A g^−1^. The device delivers specific capacities of 72.5 mAh g^−1^ (87 F g^−1^) at 0.1 A g^−1^ and 57.3 mAh g^−1^ (68.7 F g^−1^) at 1.0 A g^−1^, retaining 13.2 mAh g^−1^ (15.8 F g^−1^) at 20 A g^−1^, which highlights its exceptional rate performance. The GCD curves of LIC in Figs. 6d and S27 present a symmetric triangular shape, indicating excellent reversibility and high Coulombic efficiency, which also demonstrates good capacitance behavior. Exceptional cycle life is demonstrated by this LIC, with 73.7% capacity retention after 20,000 cycles at 5.0 A g^−1^ (Fig. 6e). The Ragone plot for the as-fabricated LIC is displayed in Fig. 6f. Ragone-type performance is achieved by this LIC, which reaches 197.9 Wh kg^−1^ at 250 W kg^−1^ (total mass normalized). Even at 50 kW kg^−1^, the system sustains an energy density of 55.5 Wh kg^−1^. Impressively, during a 60-s cycling interval, the device delivers 135.5 Wh kg^−1^, underscoring its capability for rapid energy storage and delivery. The Ragone plot further demonstrates that the energy and power densities of this LICs based on DRX-Li_x_TiOF_2_ electrode are higher than literature values for symmetrical EDLCs and state-of-the-art LICs based on battery-type negative electrodes (e.g., Li_4_Ti_5_O_12_ [67]) and pseudocapacitive negative electrodes (Nb_2_O_5_ [68], TiO_2_ [69], H_2_Ti_6_O_13_ [70], MXene [71], MoS_2_ [72]), as well as close to LICs based on graphite negative electrode (Fig. 6e). ​ We also assembled full cells with the configuration of DRX-Li_x_TiOF_2_//NMC811 and conducted systematic electrochemical performance tests, including rate capability and long cycle stability (Fig. S28). Specifically, it maintains a high reversible capacity of 187.4 mAh g^−1^ at 0.1 C and still retains 43.8 mAh g^−1^ at 10 C. For long cycle stability, the full cell exhibits remarkable durability with 81.4% capacity retention after 1000 cycles at 2 C, accompanied by a coulombic efficiency close to 100%.

Overall, the results above establish that DRX-Li_x_TiOF_2_ exhibits electrochemical and structural features of a pseudocapacitive material, despite Li^+^ intercalating into the microscale bulk. In particular, the electrochemical features are quasi-rectangular CV profile, linear charge/discharge curves and a linear dependence of the peak current on the sweep rate. Such behavior indicates intercalation pseudocapacitance rather than surface redox pseudocapacitance which is further supported by the analysis of redox site distribution. In this case, measurement of the BET surface area yielded a value of 5.74 m^2^ g^−1^ (Fig. S29) with no apparent change on cubic morphology of active materials after intercalation; thereby, the surface-normalized capacitance for the DRX-Li_x_TiOF_2_ electrode was calculated to be approximately 10.2 mF cm^−2^ at full-lithiated stage. Assuming redox contributions completely from c redox reactions of surface Ti atom (only surface pseudocapacitance), the charge number per surface Ti approach an amazing value of 97.1 e^−^ in case of 10.2 mF cm^−2^ of charge. The chemically unrealistic value rules out diffusion-controlled behavior, thereby supporting intercalation pseudocapacitance as the principal mechanism. Based on this premise, the observed capacity of 310 mAh g^−1^ translates to a redox level of 1.19 electrons per Ti atom, a reasonable value consistent with the results above on the valence state of Ti determined by XPS, EXAFS and EELS spectra. More importantly, the structure of DRX-Li_x_TiOF_2_ electrode does not undergo phase transformations during Li^+^ intercalation/deintercalation. This behavior establishes a key guideline for the design of intercalation pseudocapacitive materials.

## Conclusion

​In summary, we reported a DRX-Li_x_TiOF_2_ electrode material produced through an electrochemical driven phase transformation of cubic TiOF_2_. Throughout reversible lithiation and delithiation, DRX-Li_x_TiOF_2_ manifests the key features of a pseudocapacitive material. Such electrochemical signatures are characteristic of intercalation pseudocapacitance, which is indicated by the structural features of no phase transition during lithiation/delithiation and electrochemical features of quasi-rectangular CV profile, linear charge/discharge curve and surface-controlled current response. The pseudocapacitive behavior of this material arises from a 3D percolating network of t–t and t–o–t pathways revealed via ab initio calculations. Percolating pseudocapacitance enables robust and fast Li^+^ charge storage over a wide working potential range (0.1–2.0 V vs. Li^+^/Li). In summary, this work establishes DRX-Li_x_TiOF_2_ as a new family of pseudocapacitive materials capable of extending the operating potential beyond traditional pseudocapacitive materials (e.g., T-Nb_2_O_5_ and TiO_2_(B)) and delivering higher power than commercial intercalation materials (e.g., graphite), thereby opening a promising avenue to fabricate next-generation EES with the advantages in high safe, fast charge and high energy.

## Supplementary Information

Below is the link to the electronic supplementary material.

Supplementary file1 (DOCX 47540 KB)
