# Supplementary material for: Cation-Disordered Rock-Salt Lithium Titanium Oxyfluoride Anode Enabling High-Rate Li-Ion Storage Through a 3D Percolation Network
Source: Nanomicro Lett. 2026 Mar 10;18:277. doi: 10.1007/s40820-026-02123-w (PMC12976300; doi:10.1007/s40820-026-02123-w)
Supplement: Supplementary file 1 — Supplementary file1 (DOCX 47540 KB) [file 40820_2026_2123_MOESM1_ESM.docx]

Supporting Information for

**Cation-Disordered Rocksalt Lithium Titanium Oxyfluoride Anode Enabling High-Rate Li-Ion Storage through a 3D Percolation Network**

Jing Gao^1#^, Minghao Hua^2#^, Junze Lu^1^, Yuying Qin^3^, Shuxian Zhang^1^, Qingyu Li^1^, Lidong Yang^3^, Chengxiang Wang^1^, Xiaohang Lin^1^*, Yuanwei Sun^4^*, Longwei Yin^1^*, Rutao Wang^1^*

^1^ Shandong Provincial Key Laboratory of Electrochemical Catalysis and Conversion, School of Materials Science and Engineering, Shandong University, Ji’nan 250061, P. R. China

^2^ School of Traffic Management Engineering, Shandong Police College, Ji’nan 250014, P. R. China

^3^ Department of Industrial and Systems Engineering, The Hong Kong Polytechnic University, Hong Kong, P. R. China

^4^ Medical Science and Technology Innovation Center & Electron Microscopy Center, Shandong First Medical University & Shandong Academy of Medical Sciences, Ji’nan 250117, P. R. China

#Jing Gao and Minghao Hua contributed equally to this work.

*Corresponding authors. E-mail: [rtwang@sdu.edu.cn](mailto:rtwang@sdu.edu.cn) (Rutao Wang); [yinlw@sdu.edu.cn](mailto:yinlw@sdu.edu.cn) (Longwei Yin); [lxh12345@sdu.edu.cn](mailto:lxh12345@sdu.edu.cn) (Xiaohang Lin); [ywsun@sdfmu.edu.cn](mailto:ywsun@sdfmu.edu.cn) (Yuanwei Sun)

**S1 Experimental Section**

**S1.1 Cell assembly and test**

The cathode material of the full battery is LiNi_0.8_Mn_0.1_Co_0.1_O_2_ (NCM). ​ LiNi_0.8_Mn_0.1_Co_0.1_O_2_, acetylene black and PVDF were placed in a mortar with an 8:1:1 mass ratio and ground homogeneously. The power was then transferred to a glass bottle with NMP and stirred for 6 h. The well-mixed slurry was homogenized and coated with aluminum foil. Then the film was heated at 80℃ for 8 h in the vacuum oven. The film was punched into 14 mm diameter round electrodes with an active materials mass loading of about 2.3 mg (anode to cathode capacity ratio of 1.2:1). Celgard 2400 and glass fiber membranes were used as separators. The electrode solution was 1 M LiPF_6_ in EC: DMC: EMC (1:1:1 by volume, 100 ul). All operations were performed in an Ar filled glovebox (H_2_O and O_2_ contents <0.1 ppm). The temperature was controlled at 30 ± 1 °C throughout the entire testing process. All full cells were tested under a potential window of 1.5-3.9 V.

**S1.2 Structural characterization**

**SEM:** The morphology and structure of the prepared TiOF_2_ and Li_x_TiOF_2_ were investigated by field emission scanning electron microscopy (FESEM, JSM-7800F, JEOL, Japan). The DRX- Li_x_TiOF_2_ (0<x<2) electrodes were washed with DMC, then dried naturally in glove box before sending to the SEM characterization. In order to demonstrate the structural stability of DRX- Li_x_TiOF_2_ during cycling, the surface and side morphology of the charging and discharging electrodes after different numbers of cycles (1st, 10th, 1000th) were characterized by SEM.

**TEM and AC-TEM:** The morphological and structural information of the samples was recorded using transmission electron microscopy (TEM, JEM-F200, JEOL, Japan) and spherical aberration corrected transmission electron microscope (AC-TEM, JEM-ARM300F2). Spreading the powdered sample directly onto the microgrid in the glove box and then sent to the TEM stage.

**In-situ XRD and Ex-situ XRD:** Ex-situ X-ray diffraction (XRD, DMAX-2500PC, Japan) of Cu-Kα radiator was performed on the DRX-Li_x_TiOF_2_ electrodes to obtain structural information of the samples (C paper was chosen as a current collector for the XRD tests in order to avoid the influence of the diffraction peaks of the Cu current collector).

In-situ XRD measurements were performed on a multifunctional X-ray diffractometer (XRD, Smartlab 9 KW, Rigaku, Japan) with 2θ values ranging from 10° to 90° in the first two cycles and a scan rate of 10°min^-1^. The in-situ cell was assembled in an Ar-filled glove box (H_2_O and O_2_ contents <0.1 ppm). The in-situ cell device was equipped with a high-purity beryllium (Be) window which is used as a current collector. First, as-prepared TiOF_2_, acetylene black, and PVDF with a 7:2:1 mass ratio in NMP were mixed with a mortar. The well-mixed slurry was uniformly coated onto the Be window. The mass of the dry Be window minus the mass of the pure Be window is the mass of the coating materials. Glass fiber and Celgard 2400 were used as separators in the cell assembly. A Li metal foil (1 mm thick, 10 mm diameter) was used as the anode in the cell. The cell was discharging to 0.1 V, then charging to 2.0 V. The charge/discharge current density was set to 0.07 A g-1 during in-situ XRD.

**Inductively coupled plasma optical emission spectroscopy (ICP-AES):** The stoichiometry of the as-synthesized DRX- Li_x_TiOF_2_ was determined by inductively coupled plasma optical emission spectroscopy (EA, Vario EL cube, Germany).

**X-ray photoelectron spectroscopy (XPS):** The cycled DRX-Li_x_TiOF_2_ half-cell was disassembled in an argon-filled glovebox. The DRX-Li_x_TiOF_2_ electrode was washed with DMC, then dried. The DRX- Li_x_TiOF_2_ were vacuum sealed and stored in a glove box until XPS testing (XPS, AXIS Supra, UK).

**X-ray absorption spectroscopy (XAS):** Ti K-edge XAFS analyses were performed with Si(111) crystal monochromators at the BL14W Beam line at the Shanghai Synchrotron Radiation Facility (SSRF) (Shanghai, China). Before the analysis at the beamline, samples were placed into aluminum sample holders and sealed using Kapton tape film. The XAFS spectra were recorded at room temperature using a 4-channel Silicon Drift Detector (SDD) Bruker 5040. Ti K-edge extended X-ray absorption fine structure (EXAFS) spectra were recorded in transmission mode. Negligible changes in the line-shape and peak position of Ti K-edge XANES spectra were observed between two scans taken for a specific sample. The XAFS spectra of these standard samples were recorded in transmission mode. The spectra were processed and analyzed by the software codes Athena.

The XAFS data were processed according to the standard procedures using the Athena module implemented in the IFEFFIT software packages. The EXAFS spectra were obtained by subtracting the post-edge background from the overall absorption and then normalizing with respect to the edge-jump step. Subsequently, the χ(k) data of were Fourier transformed to real (R) space using a hanning windows (dk = 1.0 Å^−1^) to separate the EXAFS contributions from different coordination shells. To obtain the quantitative structural parameters around central atoms, least-squares curve parameter fitting was performed using the ARTEMIS module of IFEFFIT software packages.

**Grazing-incidence synchrotron X-ray diffraction:** The room temperature SXRD patterns were recorded at BL14B of the Shanghai Synchrotron Radiation Facility (SSRF) at a wavelength of 0.6888 Å. A Pilatus 2M image plate detector was employed for the data collection.

**Brunauer-Emmett-Teller (BET):** An ASAP 2020 volumetric adsorption analyzer (Micromeritics, USA) at 77 K was used to achieve the pore structure data of the samples.

**S1.3 Computational Methods**

Cluster Expansion (CE) Model: The cluster expansion technique is a lattice model that which sites can be occupied by multiple species. This approach can efficiently compute total energies of large systems in various states of order or atomic configuration and has been applied to study the cation order in DRX [S1, S2]. The cluster-expansion Hamiltonian was generated in the chemical space of Li-Ti-O-F, with pair interactions up to 7.5 Å, triplet interactions up to 6.0 Å, and quadruplet interactions up to 4.5 Å based on the rock-salt structure. Li/Ti disordering among all cation sites, and O/F disordering within anion sites were allowed. The CE model was constructed by fitting to around 1304 lattice-mapped structures and their DFT calculated energies. In the current work, the ICET is applied to optimize the CE Hamiltonian of the Li-Ti-O-F system [S3]. The effective cluster interactions (ECIs) were obtained using the Automatic Relevance Determination Regression (ARDR) algorithm. The accuracy was determined by cross-validated (CV) root mean square error (RMSE). In this work, the RMSE is 55 meV/atom, indicating good fitting and reliable prediction performances.

Monte Carlo Simulations: All canonical Monte Carlo simulations based on converged CE were performed using the mchammer module in ICET [S3]. The simulations were run in the canonical annealing mode to obtain low-energy structures. To ensure the convergence of the Li network size, 12 × 12 × 12 supercells of the MC configurations containing 3456 active sites were used. In this supercell, structure was initialized with composition Li_1152_Ti_576_O_576_F_1152_. For the connectivity analysis, the definitions of 0-TM, 1-TM, and 2-TM channels of reference were used as shown [S4]. The simulations were started from high temperatures (2000 K) and gradually approached room temperature (300 K) with a temperature step of 50 K, to ensure that the most stable arrangement can be obtained. These calculations were carried out with 2000000 steps. The last configuration at the end of each NVT simulation initialized the next simulation.

DFT Calculations: All the density functional theory (DFT) calculations with collinear spin-polarization were performed by the Vienna ab initio simulation package (VASP) using the projector augmented wave (PAW) method [S5]. The plane wave energy cutoff was set as 520 eV for the plane-wave expansions.

For cluster expansion constructing, the cells of all structures were optimized with k-points per reciprocal atom (KPPRA) of 5000. These calculations were converged to 10^−6^ eV cell^−1^ in total energy for electronic loops and 0.02 eV Å^−1^ in interatomic forces for ionic loops. For each of the structural optimization calculation, the r^2^SCAN exchange correlation function was used to better capture cation-anion hybridization and improve the accuracy of the energetics [S6]. To take into account the chemical short-range order (CSRO) property of Li_2_TiOF_2_ DRX, the representative structures in smaller supercells were created using the special quasi-ordered structures (SQoS) model [S7]. We generated representative special quasi-ordered structure (SQoS) with 3×3×3 supercell based on mimicking cluster correlations at 300 K in our MC simulations. The 216-atom DFT-available supercell (with formula Li_72_Ti_36_O_36_F_72_) was used to evaluate the final material properties.

For generating structures the delithiation structures, all possible Li-vacancy ordering in the SQoS supercell at different compositions were enumerated for obtaining the configurations with the lowest Ewald energy. Then we choose five configurations with low Ewald electrostatic energy values for DFT calculations [S8]. Due to the relatively large system, the Γ k-point was used in the reciprocal space of the Brillouin zone. The resulting lowest-energy ordering structures for each composition were then used for further analysis. The pymatgen code was utilized for the structure analysis [S9]. For every composition, the voltage with respect to Li concentration ($x_{1}$, $x_{2}$) was given by

$v=-\frac{E_{\left( x_{2} \right)}-E_{\left( x_{1} \right)}-\left( x_{2}-x_{1} \right)E_{\left( \mathrm{Na} \right)}}{ⅇ\left( x_{2}-x_{1} \right)}$ (S1)

where $E$ is the DFT total energy, and $e$ is the electronic charge [S10].

The lithium ionic probability densities were calculated from the atom trajectories monitored during the ab initio molecular dynamics (AIMD) simulations. The Li_54_Ti_36_O_36_F_72_ structures were initialized from the above mentioned DFT-relaxed ground states. All the AIMD calculations were performed in the canonical ensemble (NVT) with a time step of 2 fs and using a Nosé–Hoover thermostat for a period of 100 ps. [S11] The AIMD simulations were run at 600 K, 800 K, 1000 K, 1200 K, and 1500 K.

The Li migration barriers were calculated using the climbing image nudged elastic band (CI-NEB) method [S12]. For all NEB calculations, five linearly interpolated intermediate images were used to find the minimum energy path. The energies and forces were converged to 10^−5^ eV cell^−1^ and 0.05 eV Å^−1^, respectively. At the start, the SQoS supercell at 300 K with formula Li_72_Ti_36_O_36_F_72_ was used as starting structure for the NEB calculations. For the fully occupied Li_72_Ti_36_O_36_F_72_, one Li atom was inserted into a low-energy 0-TM site, and the barriers for the tetrahedron-tetrahedron (t-t) and tetrahedron-octahedron-tetrahedron hops (t-o-t, including opposing t-o-t, corner-sharing t-o-t, edge-sharing t-o-t) were then calculated. For the Li_72_Ti_36_O_36_F_72_ structure with a few vacant octahedral sites, a supercell with one Li vacancy was created by removing an individual Li atom from the fully occupied lattice and then re-optimizing the atomic coordinates. The barriers for the octahedron-octahedron (o-o) and octahedron-tetrahedron-octahedron (o-t-o) Li diffusion pathways were then calculated [S10]. The supercell structure with formula Li_36_Ti_36_O_36_F_72_ was attempted in NEB calculations to investigate the Li migration barrier where a large amount of Li occupies the tetrahedral sites. The structures were constructed by removing a Li atom at tetrahedral sites in the lowest energy Li_36_Ti_36_O_36_F_72_ configuration and fully relaxed.

We use the kinetically resolved activation (KRA) barrier to remove the directional dependence of Li migration [S13], which is defined as:

$E_{\mathrm{KRA}}=E_{\mathrm{barr}\dot{i}\mathrm{er}}-\frac{1}{2}\left( E_{\mathrm{ini}}+E_{\mathrm{fin}} \right)$ (S2)

where the $E_{\mathrm{barr}\dot{i}\mathrm{er}}$ is the activated transition state barrier, $E_{\mathrm{ini}}$ and $E_{\mathrm{fin}}$ are the calculated energies of the initial state and the end state structures in CI-NEB.

**S2 Supplementary Figures and Tables**


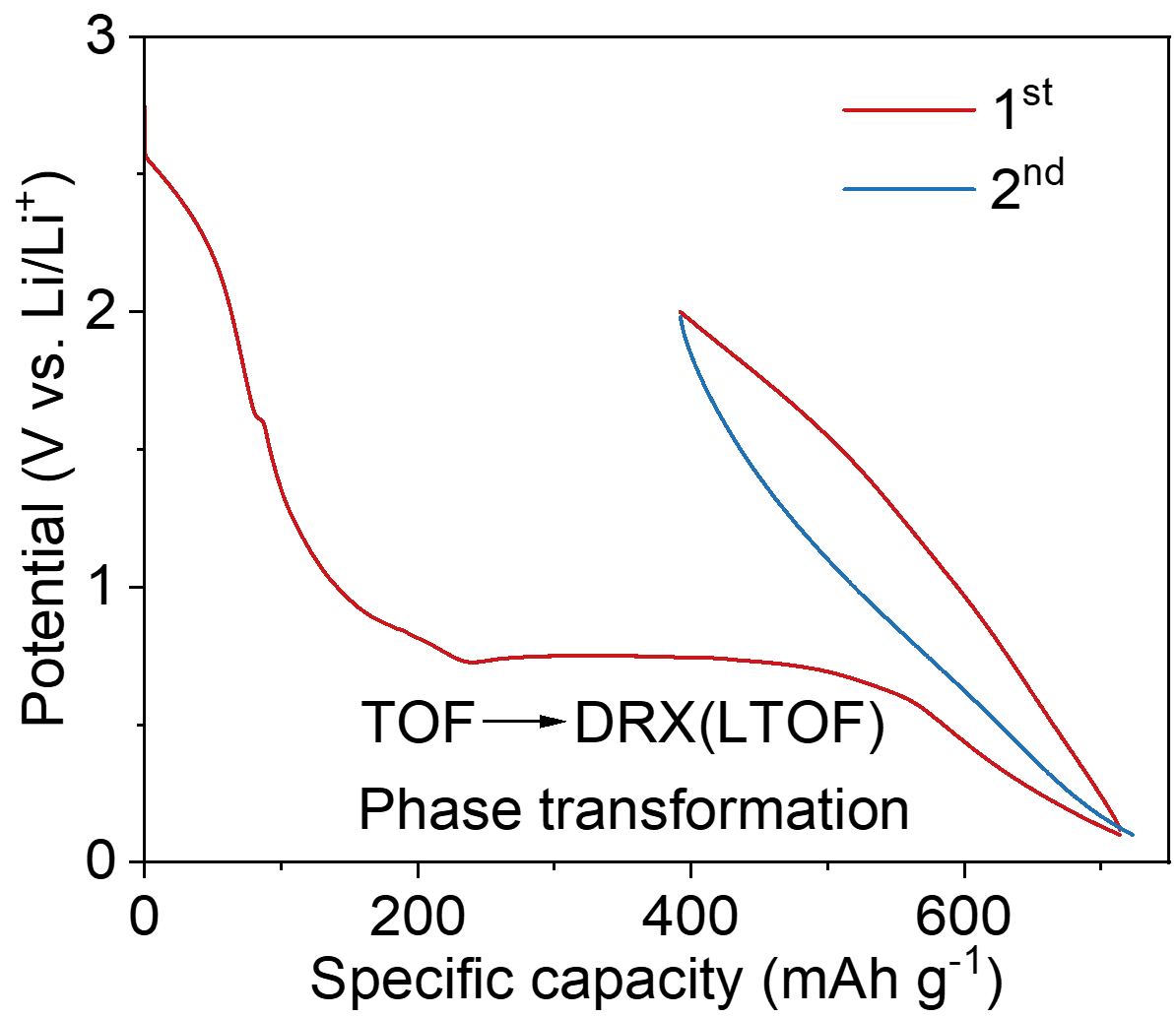


**Fig. S1** Voltage curves for the first two cylces at 0.1 A g^-1^ with a voltage window of 0.1-2.0 V


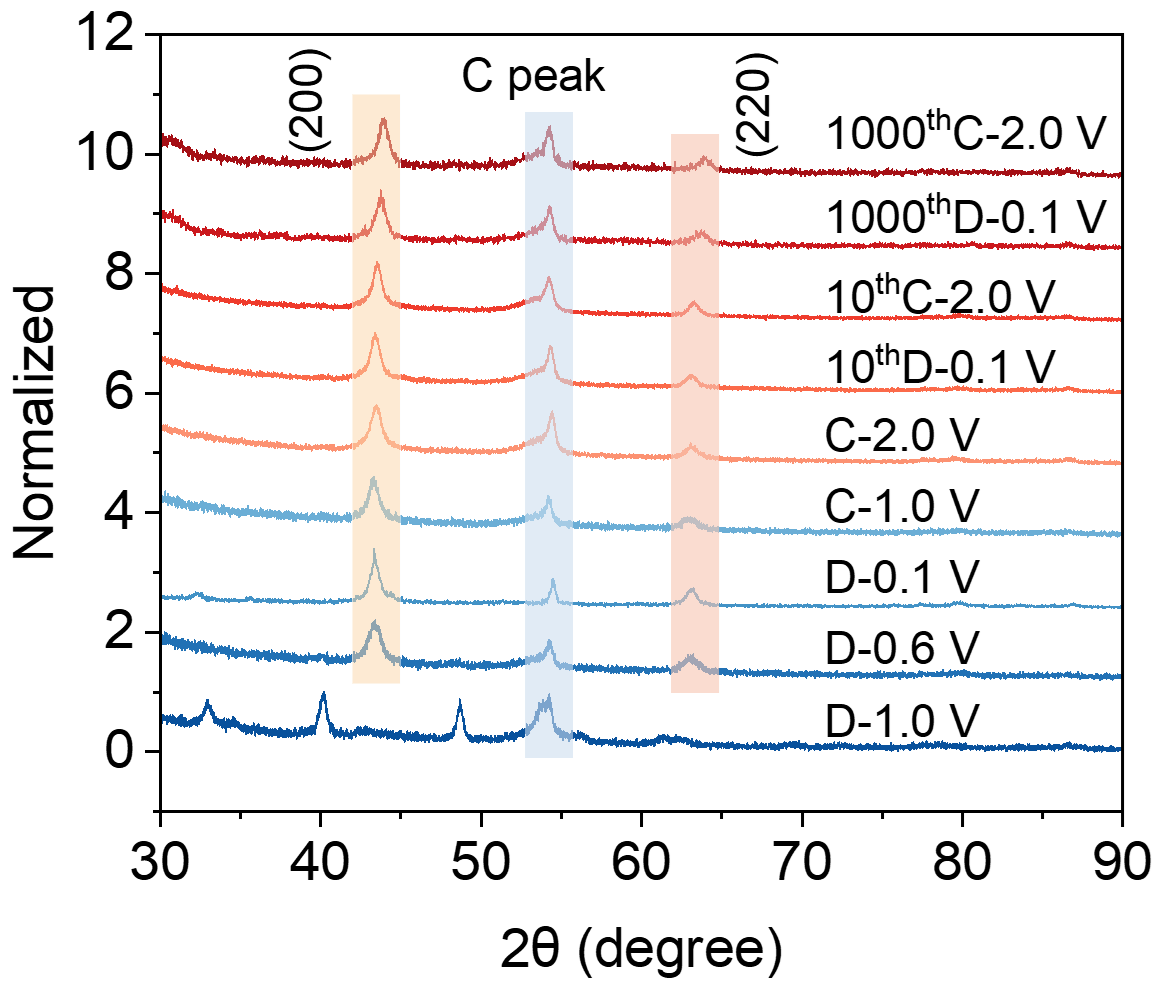


**Fig. S2** Ex-situ XRD patterns of the electrodes in different charging and discharging states


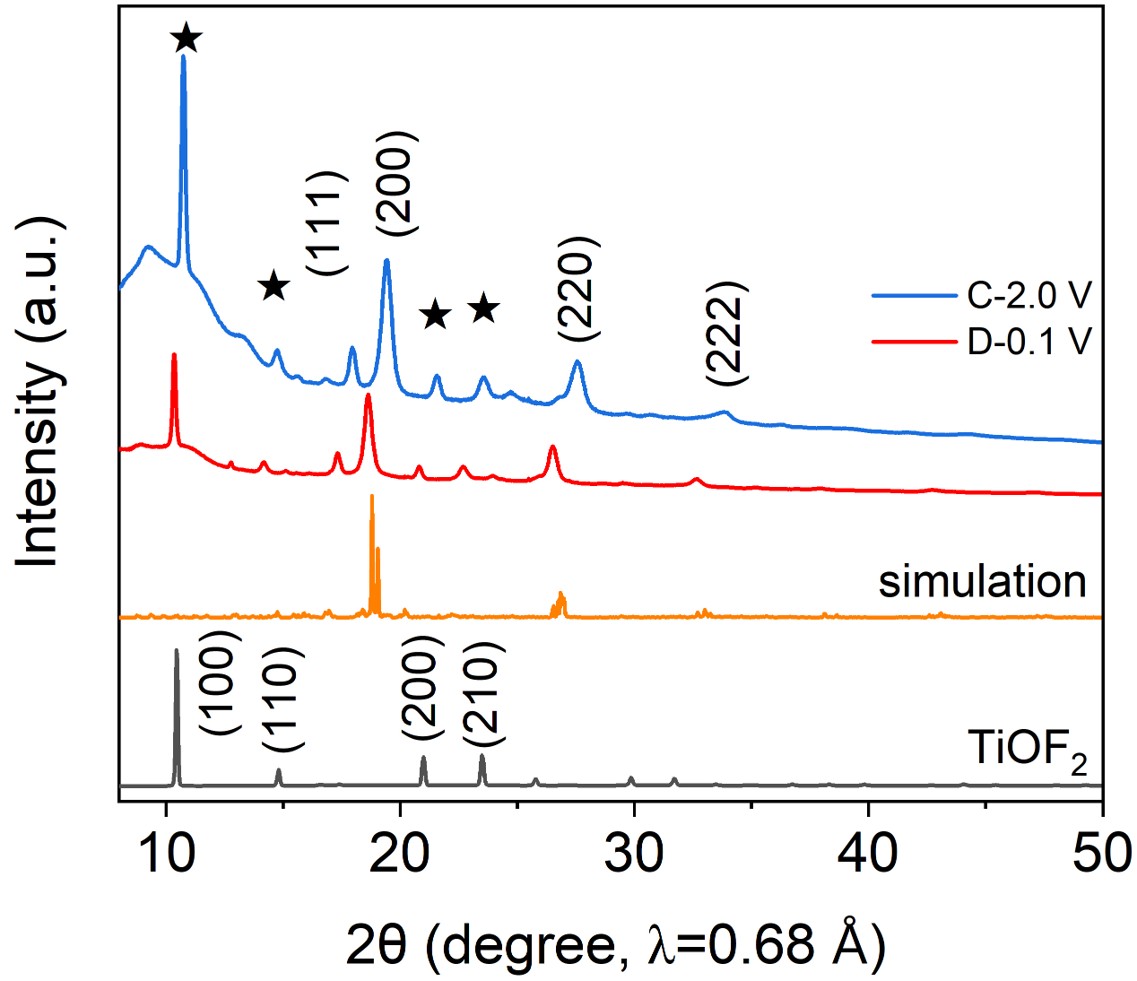


**Fig. S3** Grazing-incidence synchrotron X-ray diffraction of the D-0.1 V and C-2.0 V. Simulated XRD pattern was simulated from Monte Carlo (MC) theoretical calculations


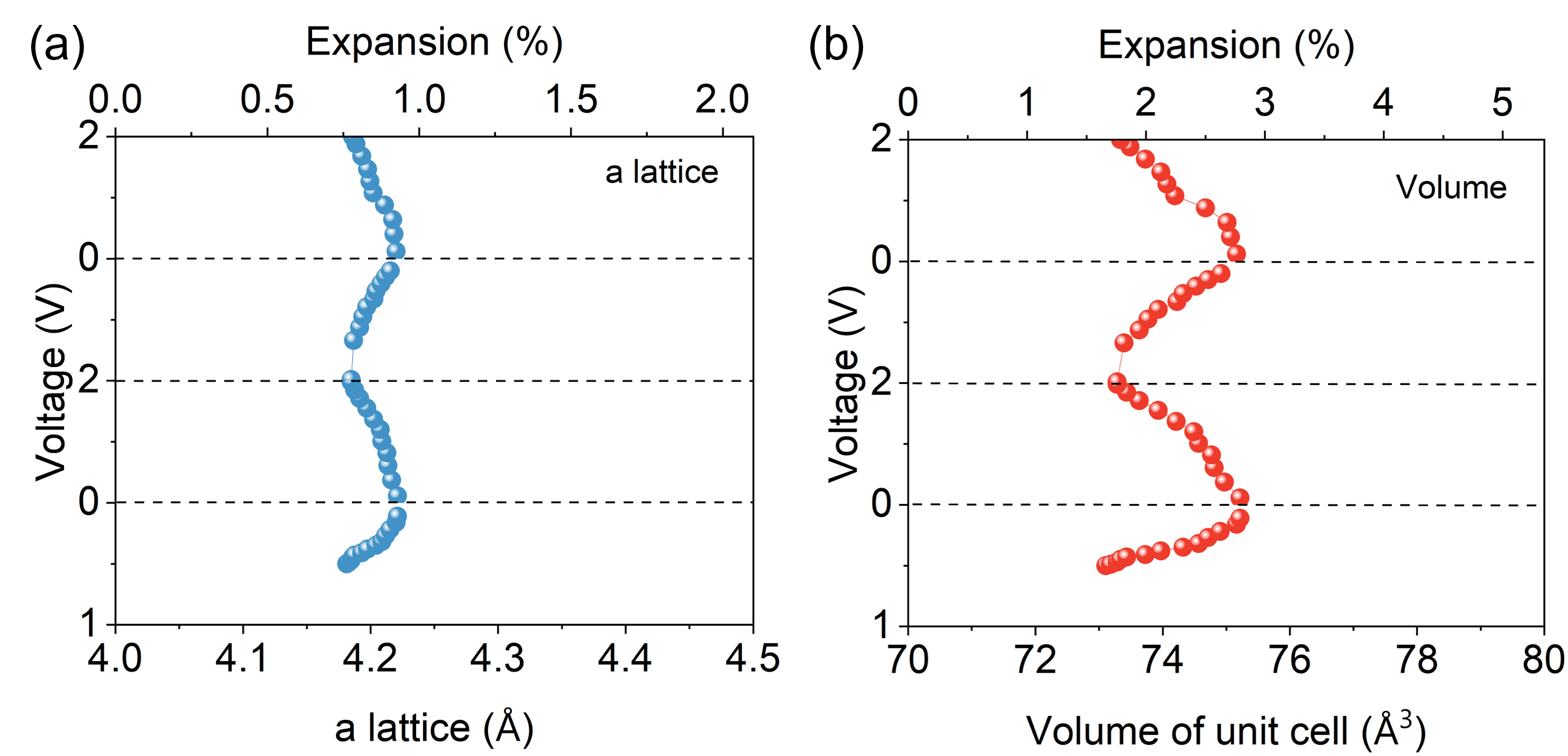


**Fig. S4** (**a**) Evolution of the lattice parameter a of DRX-Li_x_TiOF_2_. (**b**) The evolution of the unit cell volume of DRX-Li_x_TiOF_2_


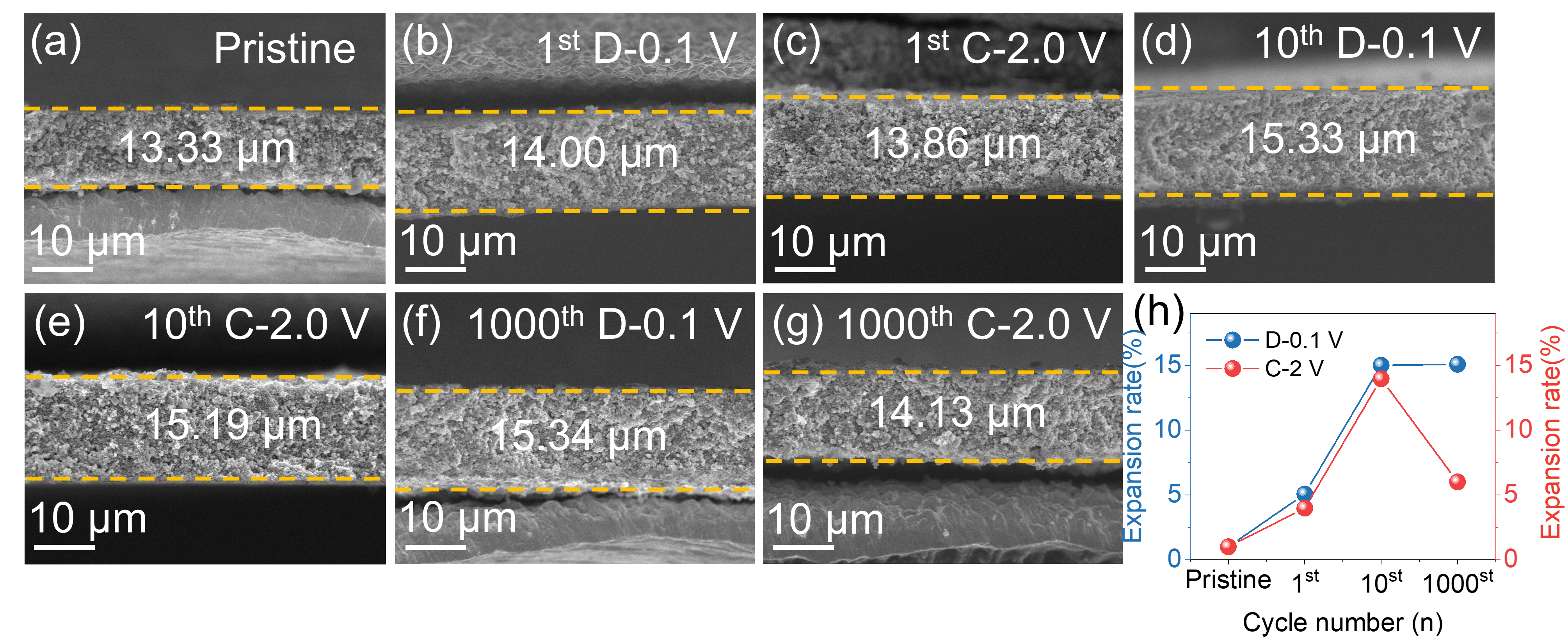


**Fig. S5** (**a**) Cross-section of a pristine TiOF_2_ electrode. (**b**) Cross-section of electrode discharged to 0.1 V. (**c**) Cross-section of electrode charged to 2.0 V. (**d**) Cross-section of electrode discharged to 0.1 V after 10 cycles. (**e**) Cross-section of electrode charged to 2.0 V after 10 cycles. (**f**) Cross-section of electrode discharged to 0.1 V after 1000 cycles. (**g**) Cross-section of electrode charged to 2.0 V after 1000 cycles. (**h**) Plot of cross-section thickness variation


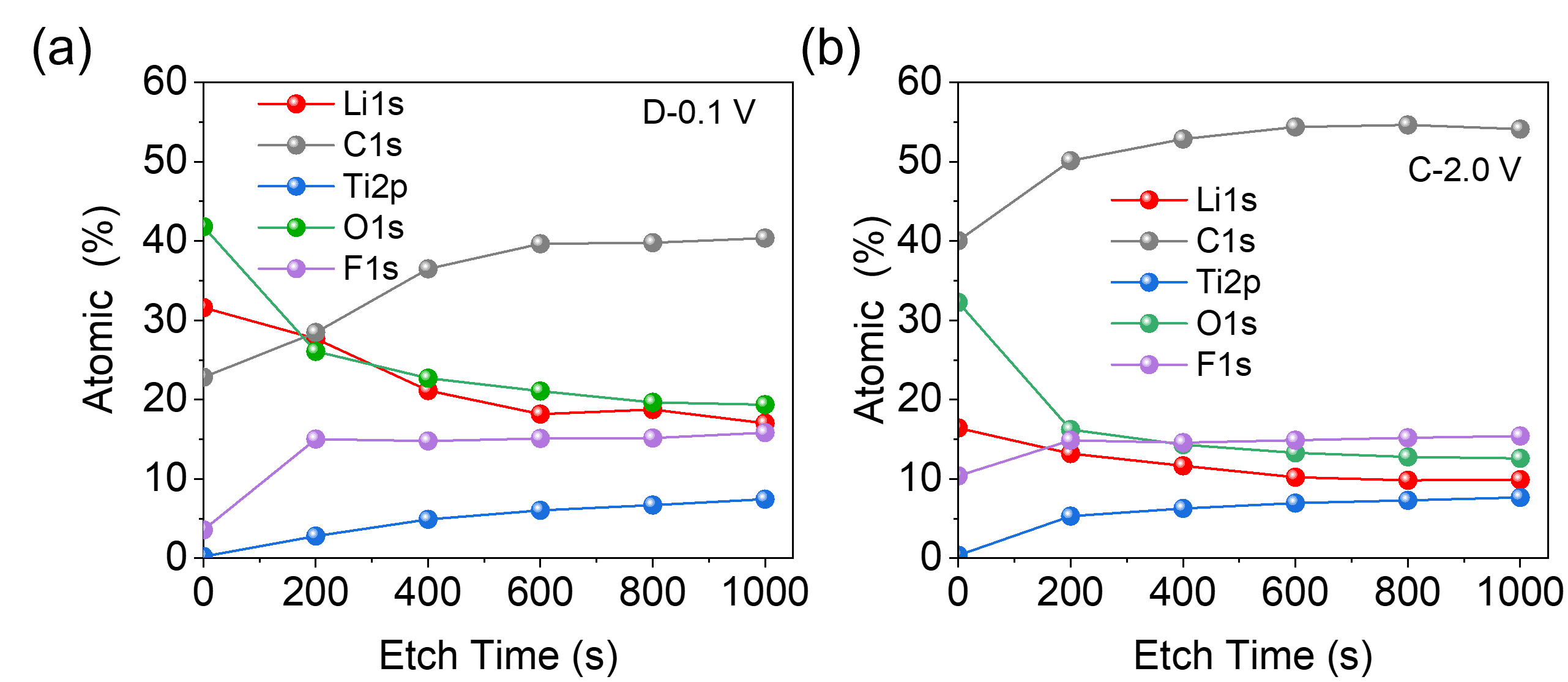


**Fig. S6** The changes in the atomic content of each element under different etching times


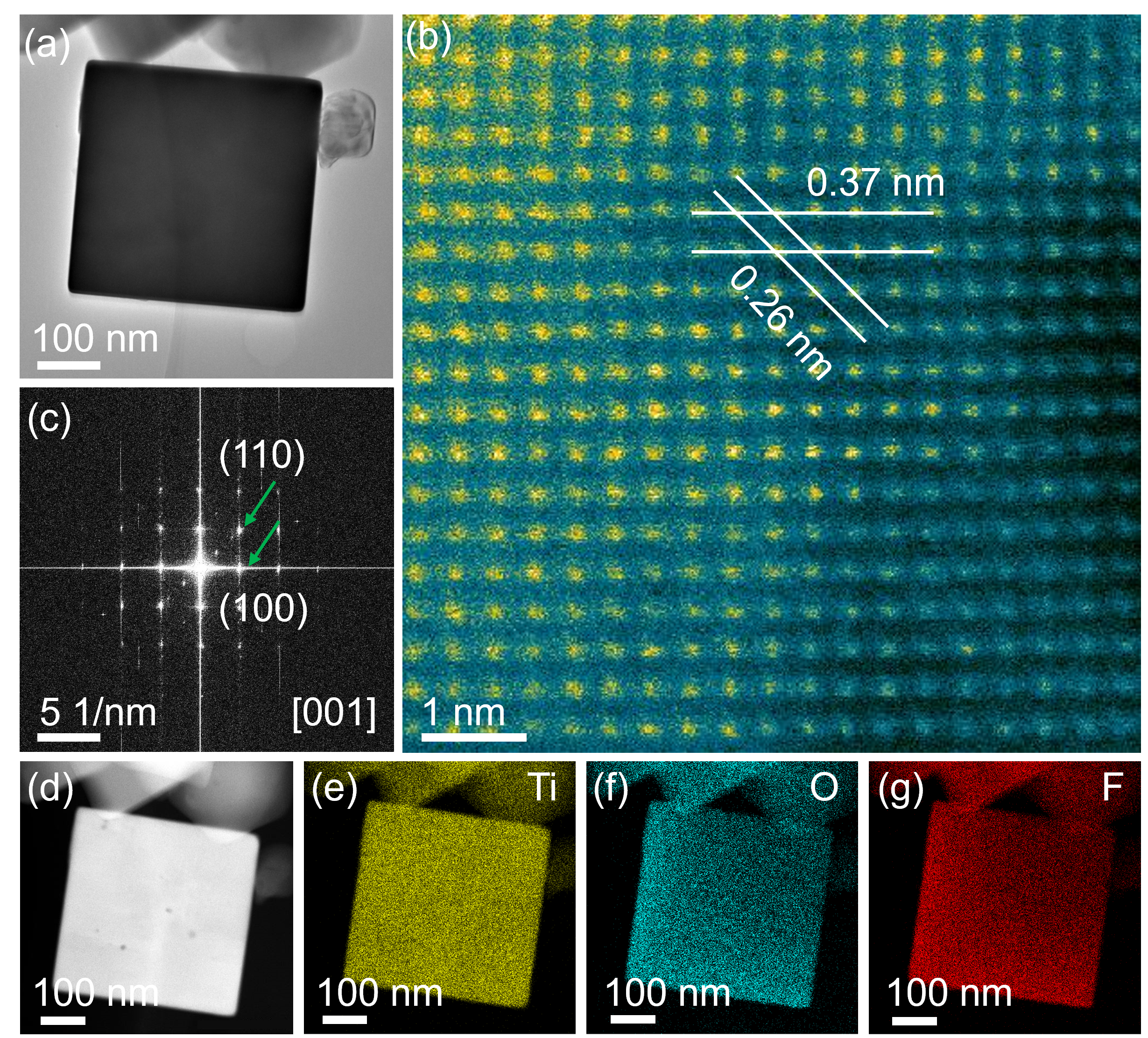


**Fig. S7** (**a**) Low resolution TEM micrograph of TiOF_2_. (**b**) HAADF-STEM image of TiOF_2_. (**c**) FFT image of TiOF_2_. (**d-g**) EDS mapping of the elemental distribution of TiOF_2_


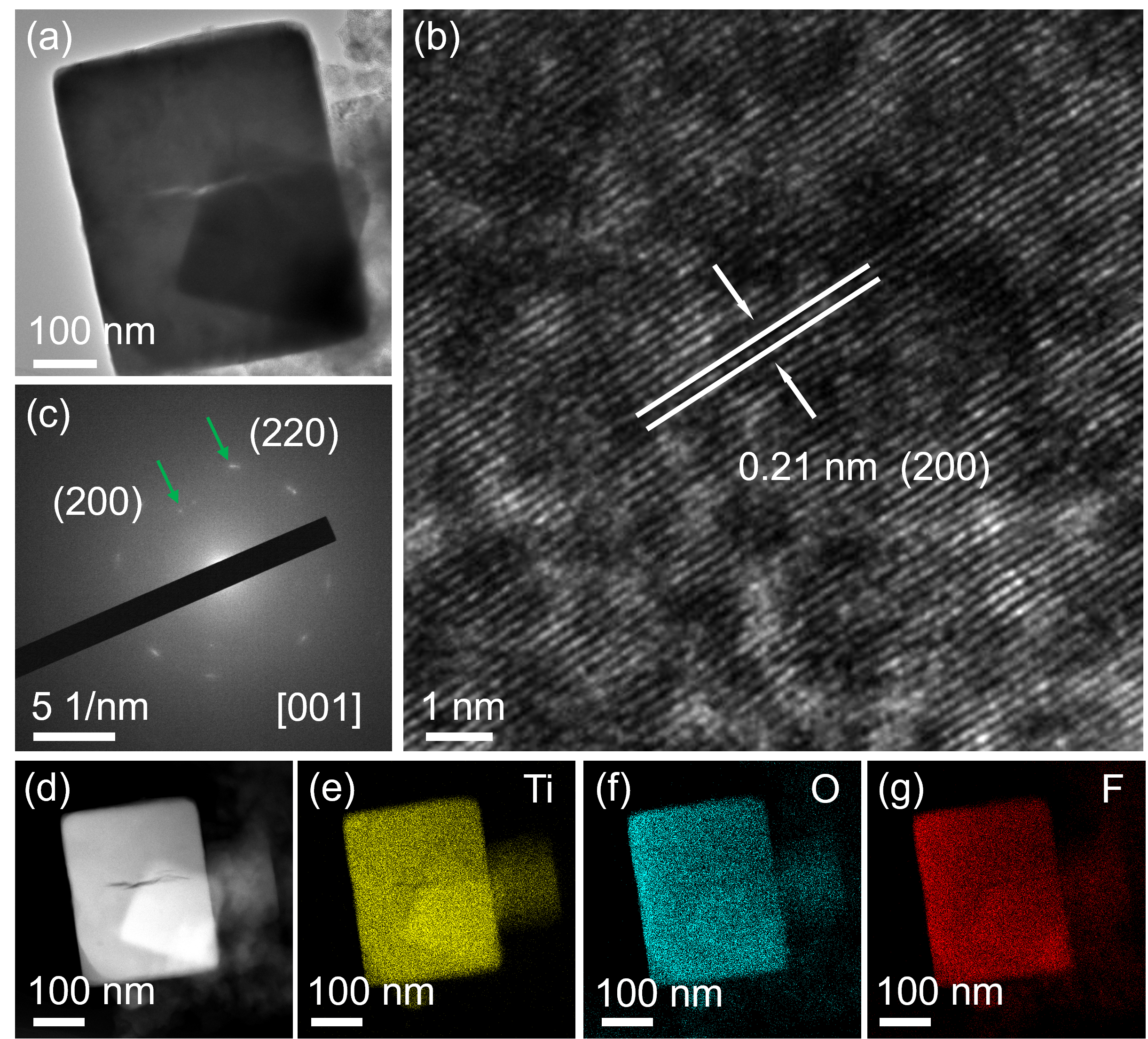


**Fig. S8** (**a**) Low resolution TEM micrograph of D-0.1 V. (**b**) High resolution TEM micrograph of D-0.1 V. (**c**) SEAD pattern of D-0.1 V. (**d-g**) EDS mapping of the elemental distribution of D-0.1 V


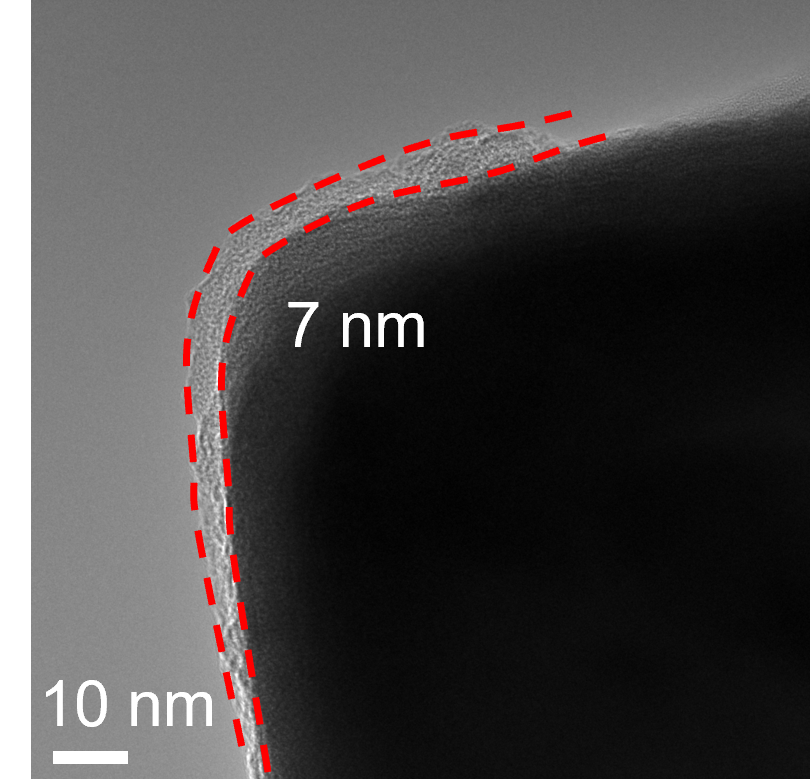


**Fig. S9** (**a, b**) TEM images of D-0.1 V on first discharge. The SEI layer is indicated by the red dashed lines


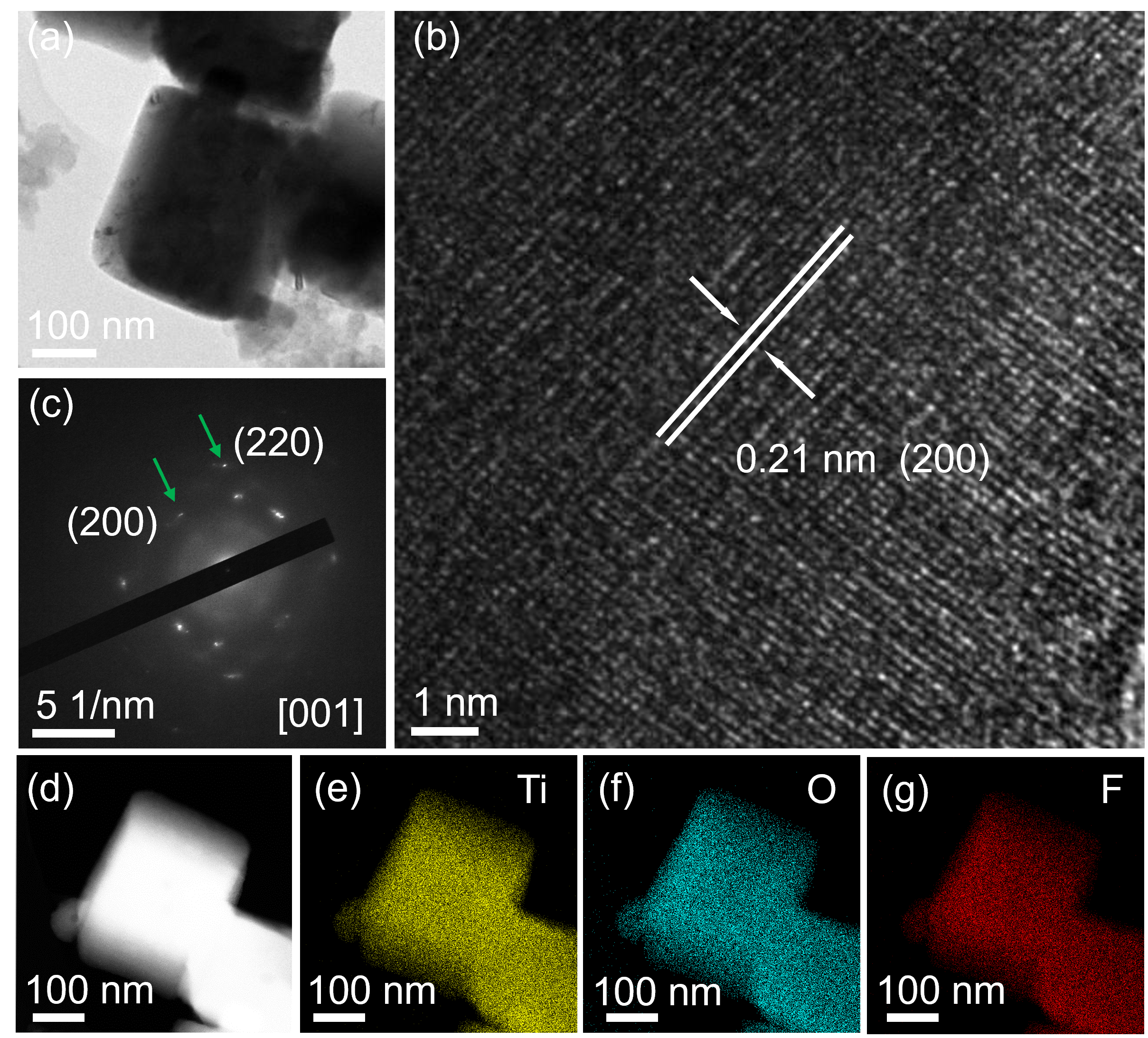


**Fig. S10** (**a**) Low resolution TEM micrograph of C-2.0 V. (**b**) High resolution TEM micrograph C-2.0 V. (**c**) SEAD pattern of C-2.0 V. (**d-g**) EDS mapping of the elemental distribution of C-2.0 V


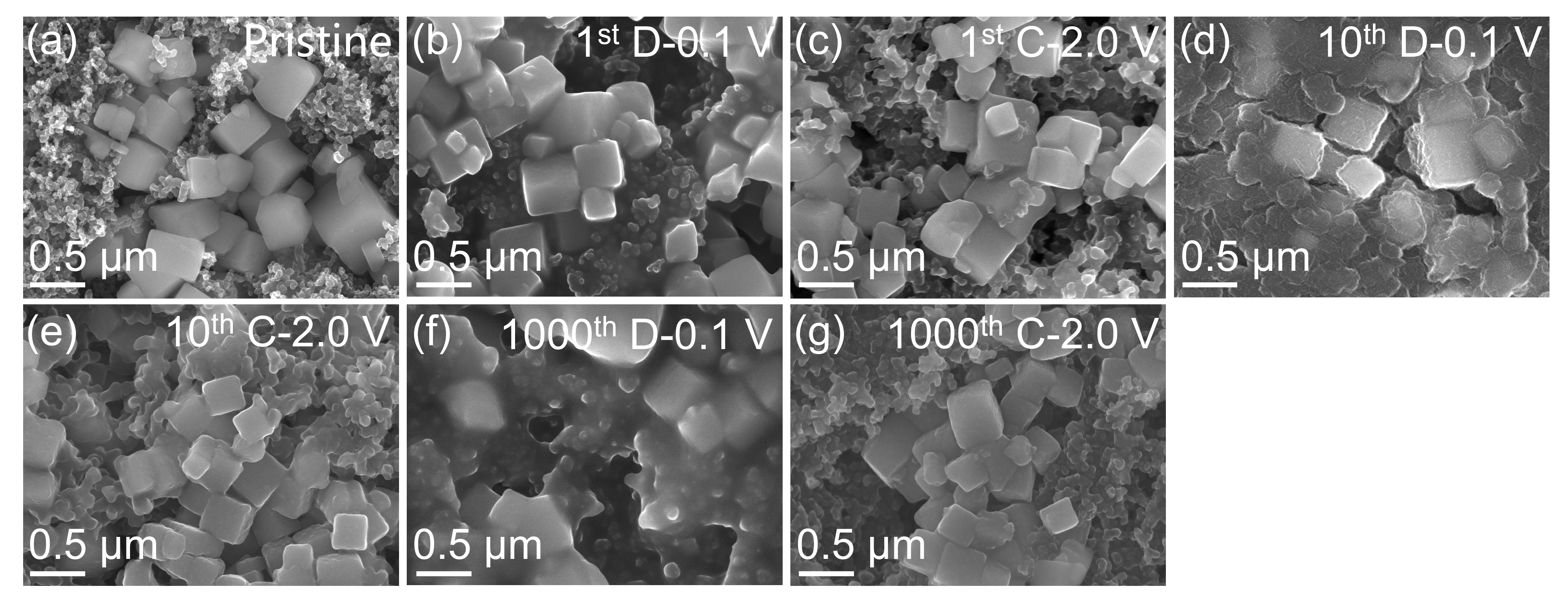


**Fig. S11** (**a**) SEM image of TiOF_2_ powder. (**b**) SEM image of electrode surface of TiOF_2_ (**c**) SEM image of the electrode surface discharged to 0.1 V. (**d**) SEM image of electrode surface charged to 2.0 V. (**e**) SEM image of electrode surface discharged to 0.1 V after 10 cycles. (**f**) SEM image of electrode surface charged to 2.0 V after 10 cycles. (**g**) SEM image of electrode surface discharged to 0.1 V after 1000 cycles. (**h**) SEM image of electrode surface charged to 2.0 V after 1000 cycles


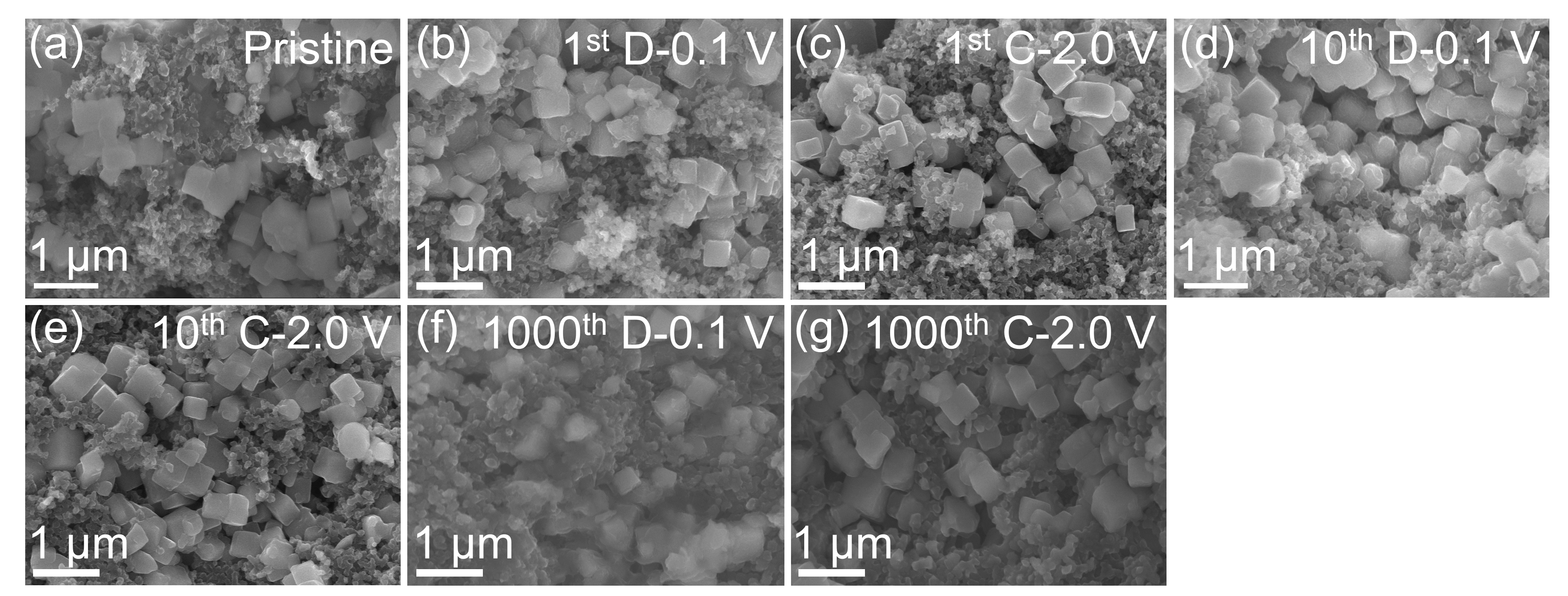


**Fig. S12** (**a**) High magnification SEM image of the cross-section of pristine TiOF_2_ electrode. (**b**) High magnification SEM image of the cross-section of electrode discharged to 0.1 V. (**c**) High magnification SEM image of the cross-section of electrode charged to 2.0 V. (**d**) High magnification SEM image of the cross-section of electrode discharged to 0.1 V after 10 cycles. (**e**) High magnification SEM image of the cross-section of electrode charged to 2.0 V after 10 cycles. (**f**) High magnification SEM image of the cross-section of electrode discharged to 0.1 V after 1000 cycles. (**g**) High magnification SEM image of the cross-section of electrode charged to 2.0 V after 1000 cycles


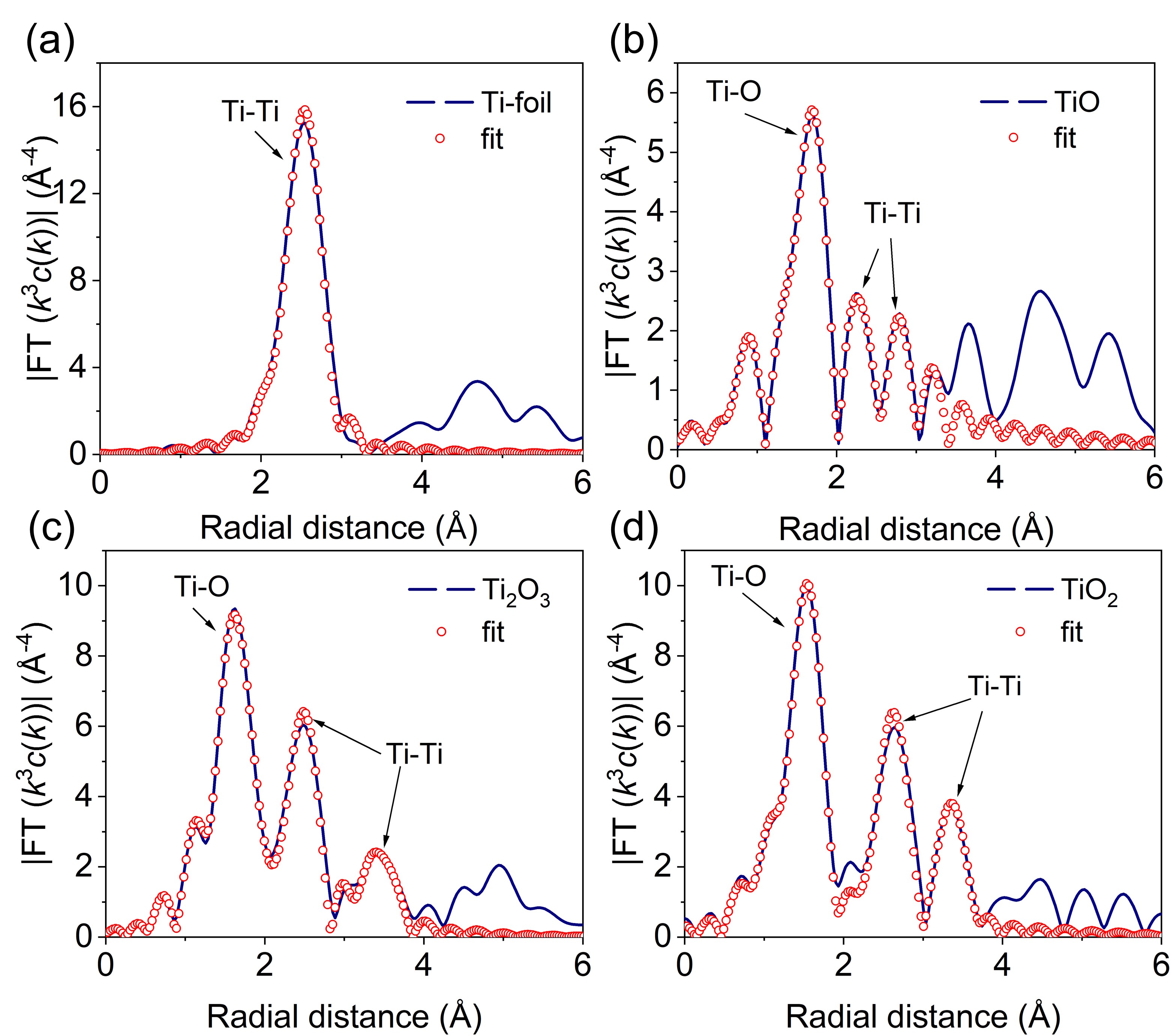


**Fig. S13** (**a**) The corresponding k^3^-weighted EXAFS fitting curves at R space for the Ti foil. (**b**) The corresponding k^3^-weighted EXAFS fitting curves at R space for the TiO. (**c**) The corresponding k^3^-weighted EXAFS fitting curves at R space for the Ti_2_O_3_. (**d**) The corresponding k^3^-weighted EXAFS fitting curves at R space for the TiO_2_

**
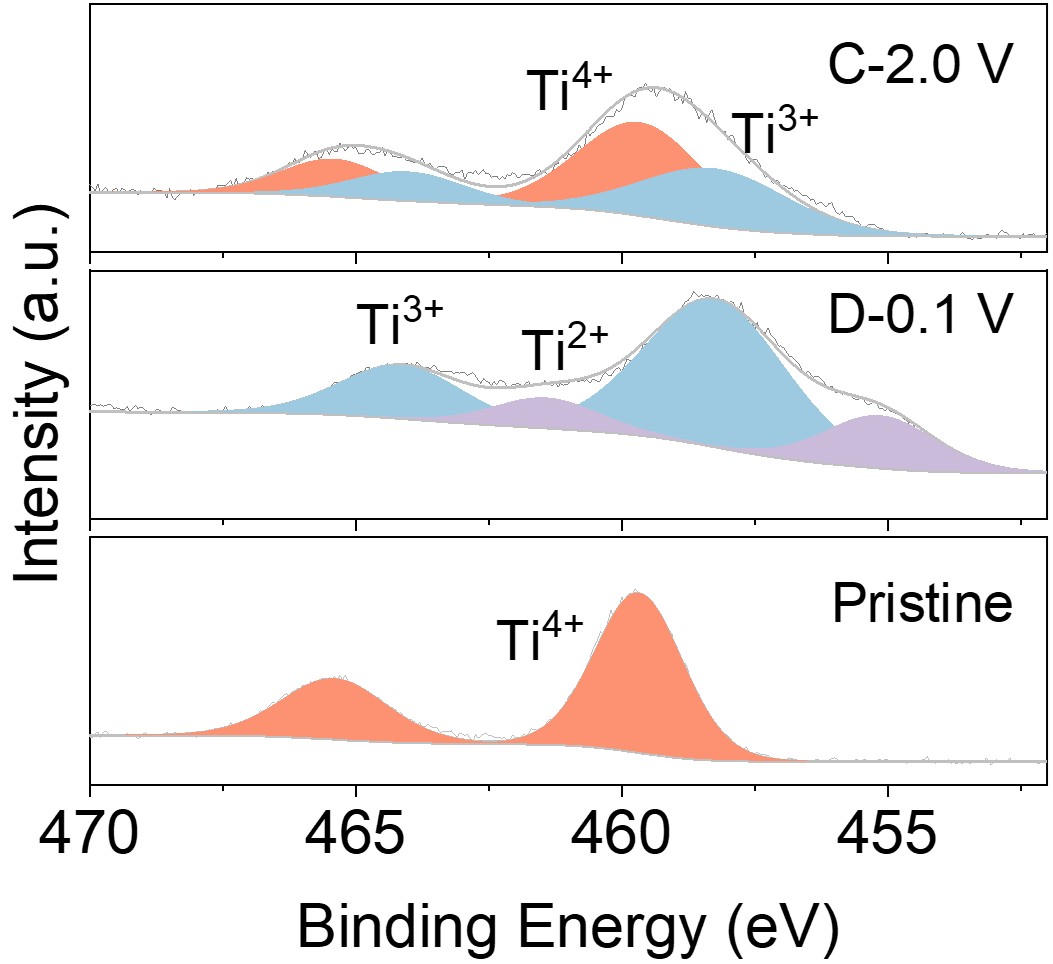
**

**Fig. S14** XPS spectra of the DRX-Li_x_TiOF_2_ electrodes upon lithiation and delithiation processes in the range of 0.1-2.0 V


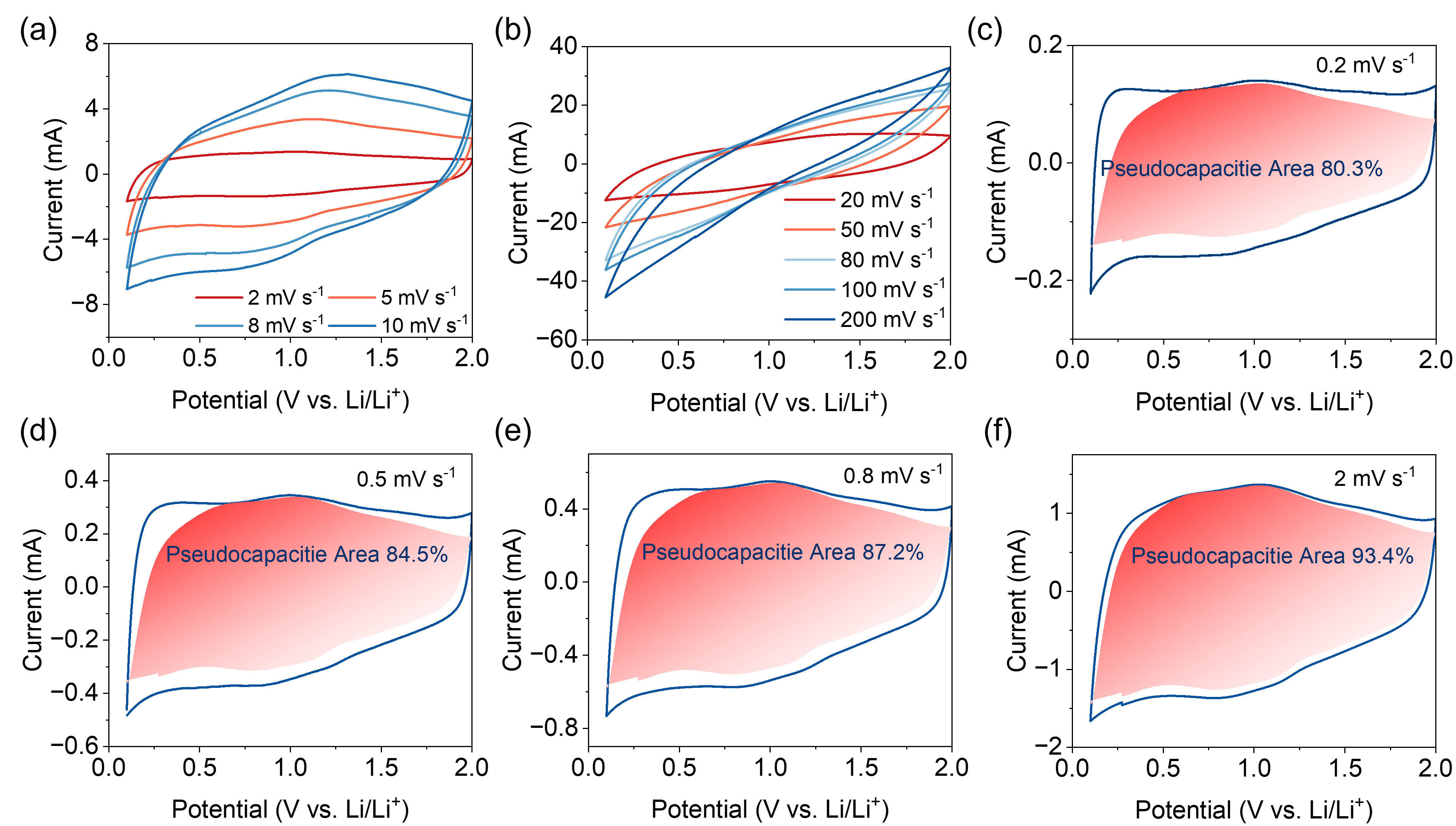


**Fig. S15** **Electrochemical performance of DRX-Li_x_TiOF_2_ electrodes with 1 mg mass loading (0.64 mg cm^-2^) of active material.** (**a**) CV curves at sweep rates varied from 2 to 10 mV s^-1^. (**b**) CV curves at sweep rates varied from 20 to 200 mV s^-1^. Capacitive contribution (red region) to the total charge storage at 0.2 mV s^-1^ (**c**) 0.5 mV s^-1^ (**d**) 0.8 mV s^-1^ (**e**) 2 mV s^-1^ (**f**)


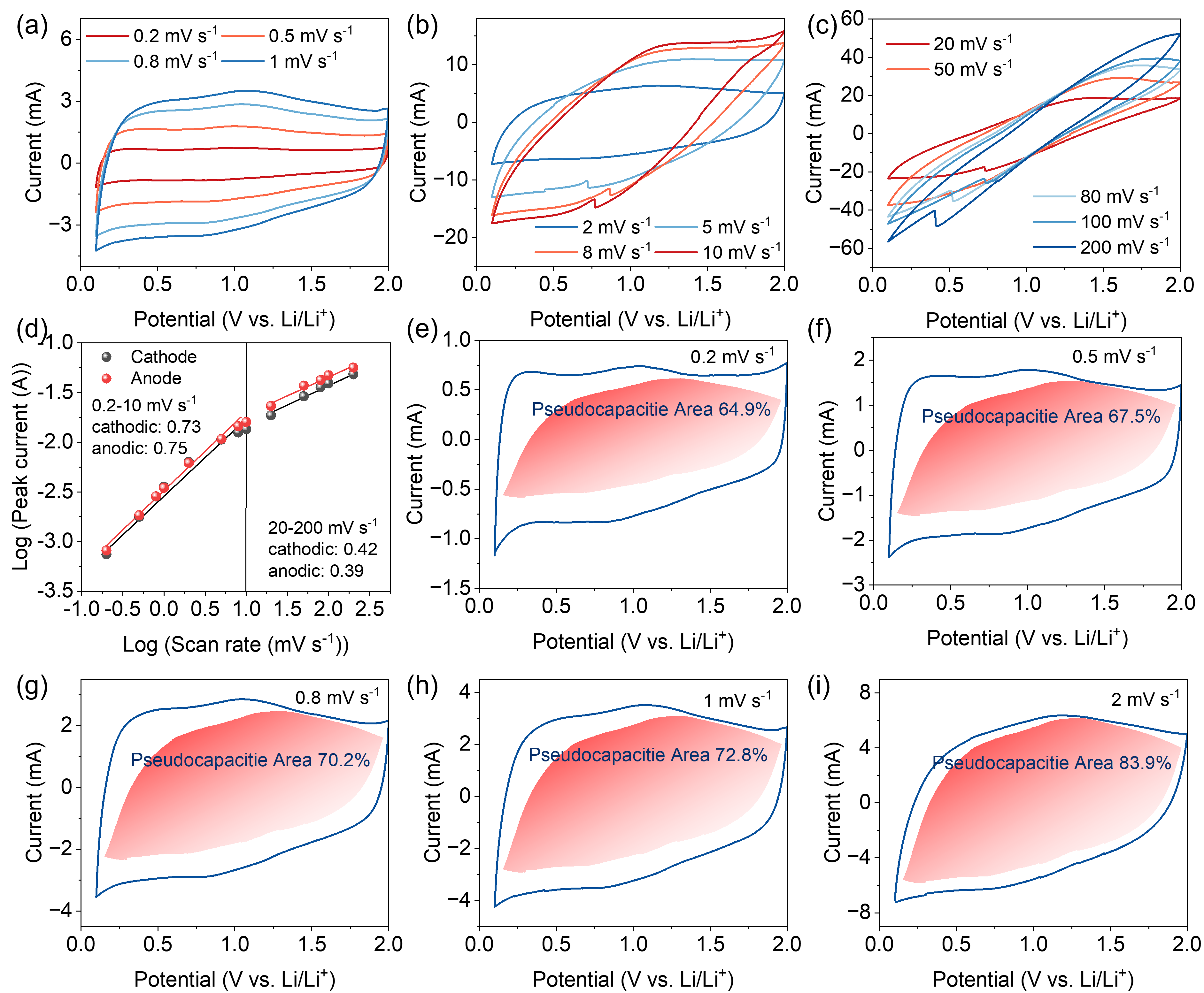


**Fig. S16 Electrochemical performance of DRX-Li_x_TiOF_2_ electrodes with 6 mg mass loading (3.9 mg cm^-2^) of active material.** (**a**) CV curves at sweep rates varied from 0.2 to 1 mV s^-1^. (**b**) CV curves at sweep rates varied from 2 to 10 mV s^-1^. (**c**) CV curves at sweep rates varied from 20 to 200 mV s^-1^. (**d**) Determination of the b values for the cathodic and anodic peak based on the plots of log(i) versus log(v). Capacitive contribution (red region) to the total charge storage at 0.2 mV s^-1^ (**e**) 0.5 mV s^-1^ (f) 0.8 mV s^-1^ (**g**) 1 mV s^-1^ (**h**) and 2 mV s^-1^ (**i**)


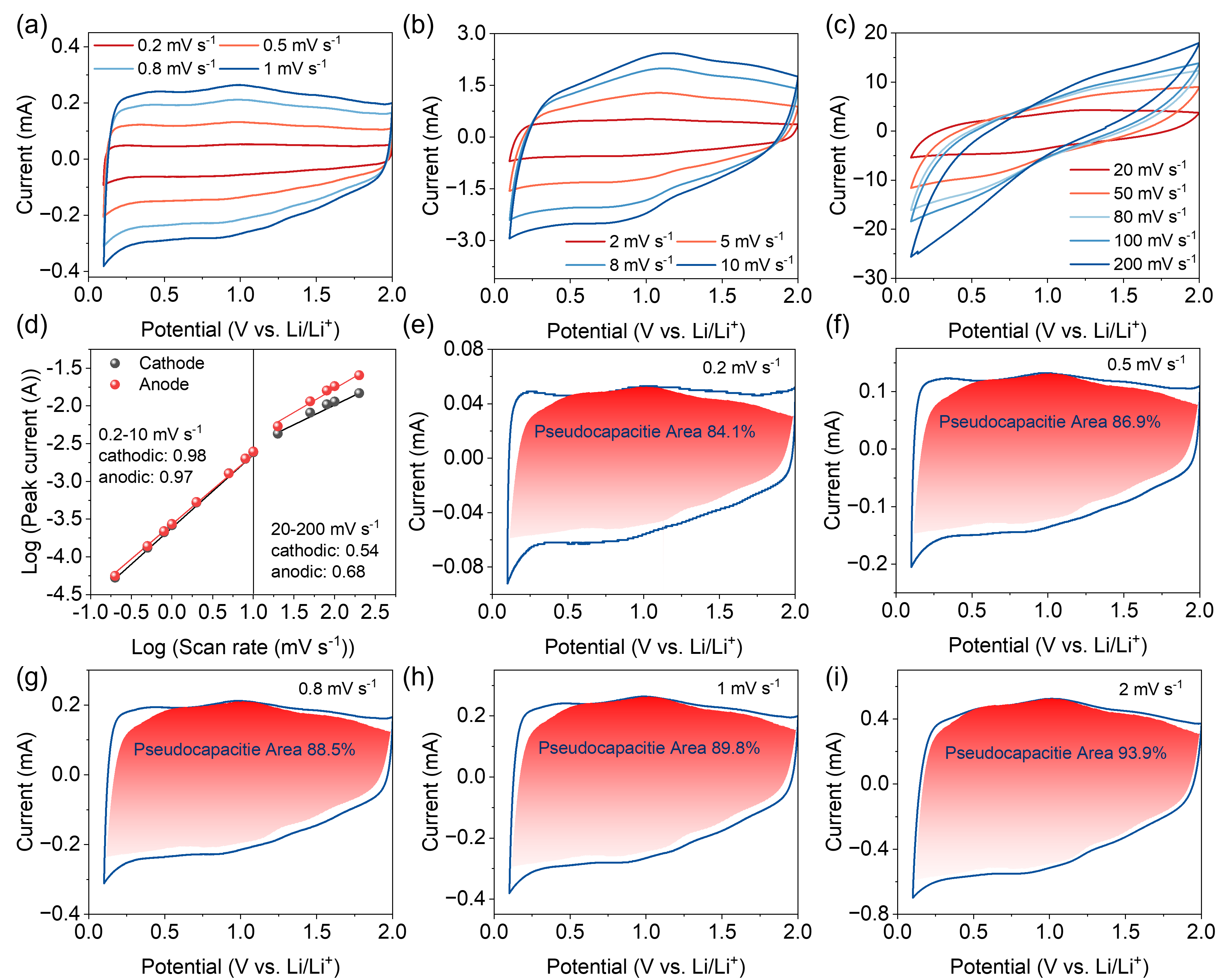


**Fig. S17** **Electrochemical performance of DRX-Li_x_TiOF_2_ electrodes with 0.39 mg mass loading (0.25 mg cm^-2^) of active material.** (**a**) CV curves at sweep rates varied from 0.2 to 1 mV s^-1^. (**b**) CV curves at sweep rates varied from 2 to 10 mV s^-1^. (**c**) CV curves at sweep rates varied from 20 to 200 mV s^-1^. (**d**) Determination of the b values for the cathodic and anodic peak based on the plots of log(i) versus log(v). Capacitive contribution (red region) to the total charge storage at 0.2 mV s^-1^ (**e**) 0.5 mV s^-1^ (**f**) 0.8 mV s^-1^ (**g**) 1 mV s^-1^ (**h**) and 2 mV s^-1^ (**i**)

**
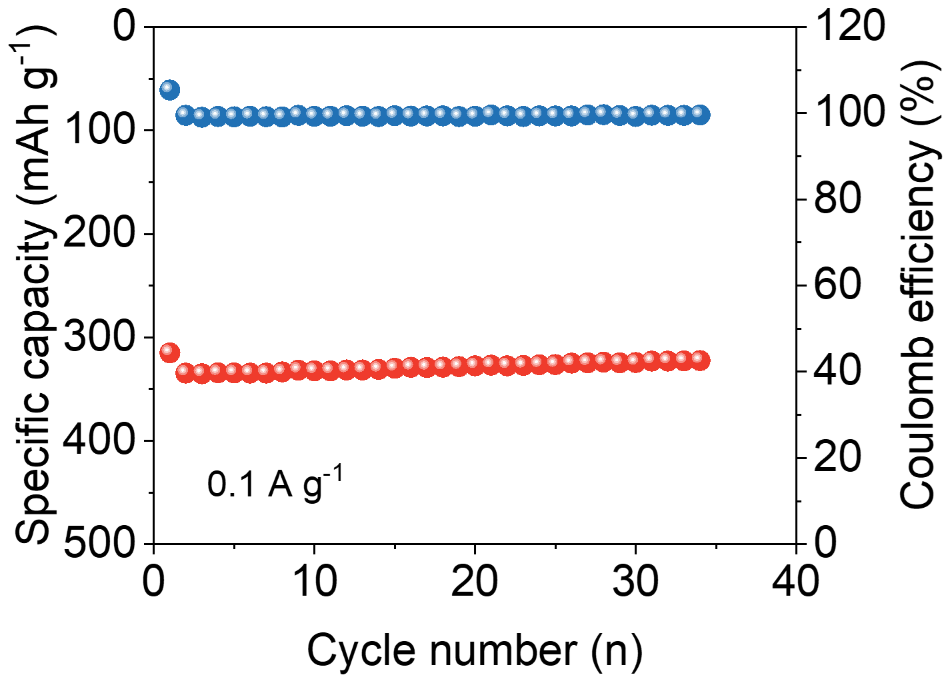
**

**Fig. S18** Long-term cycling stability for DRX-Li_x_TiOF_2_ anodes at 0.1 A g^-1^


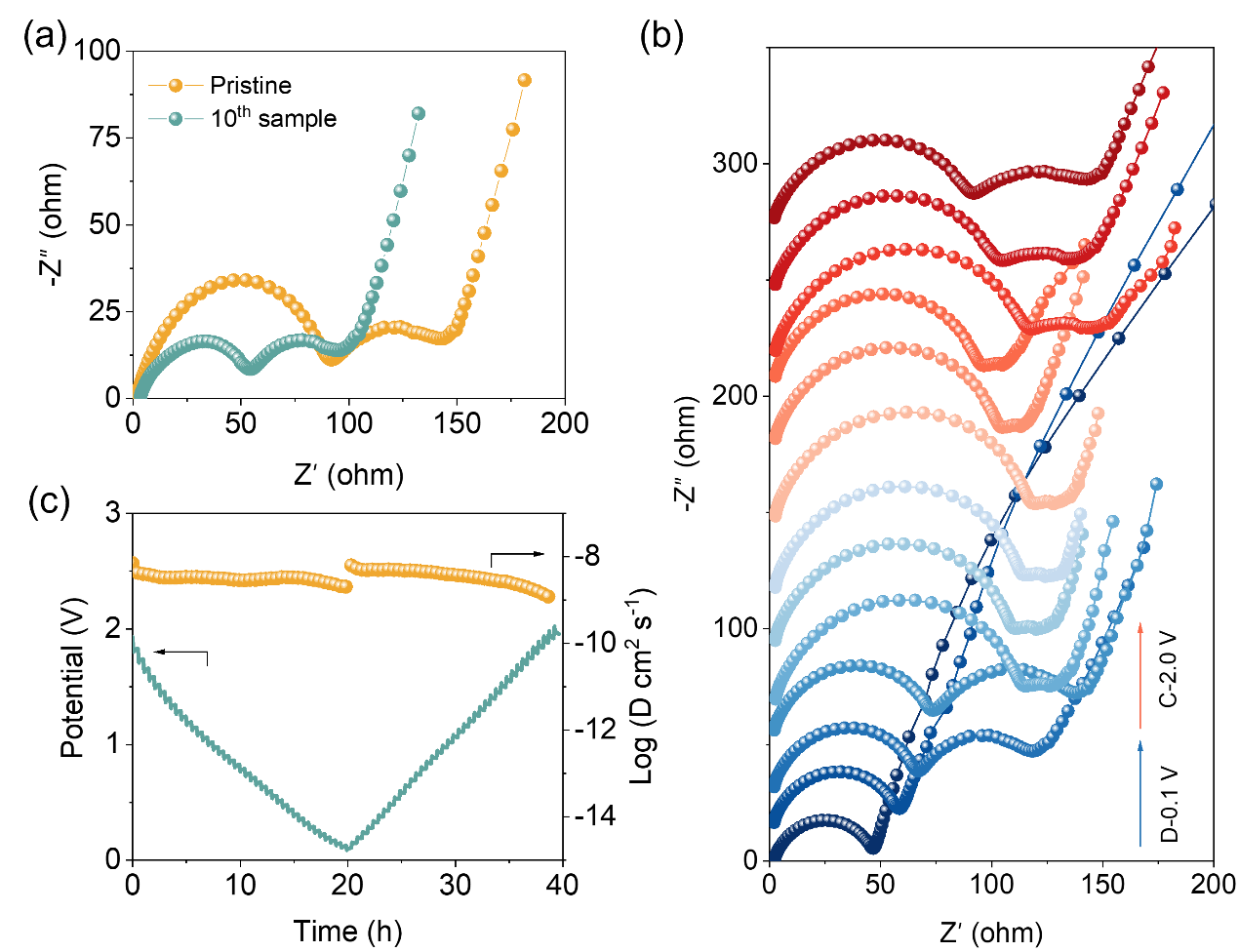


**Fig. S19** (**a**) Nyquist plot of DRX- LiₓTiOF₂ achieved at 0.1 V (vs. Li/Li^+^). (**b**) Nyquist plot of DRX-LiₓTiOF₂ at different voltage. (**c**) GITT curves and the corresponding Li^+^ diffusion coefficient


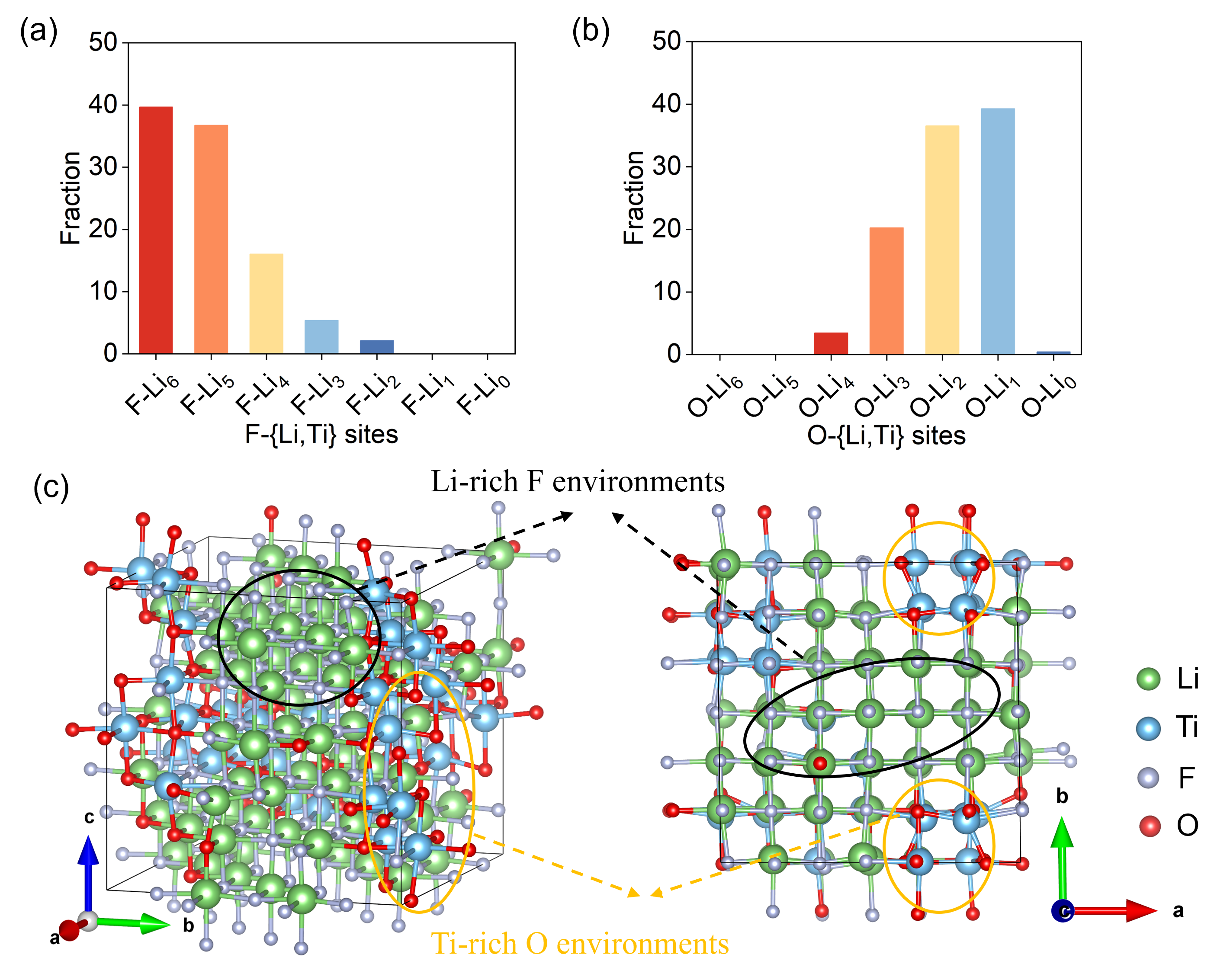


**Fig. S20** The analysis of the O/F coordination environments in pristine Li_2_TiOF_2_ MC structure at 300K


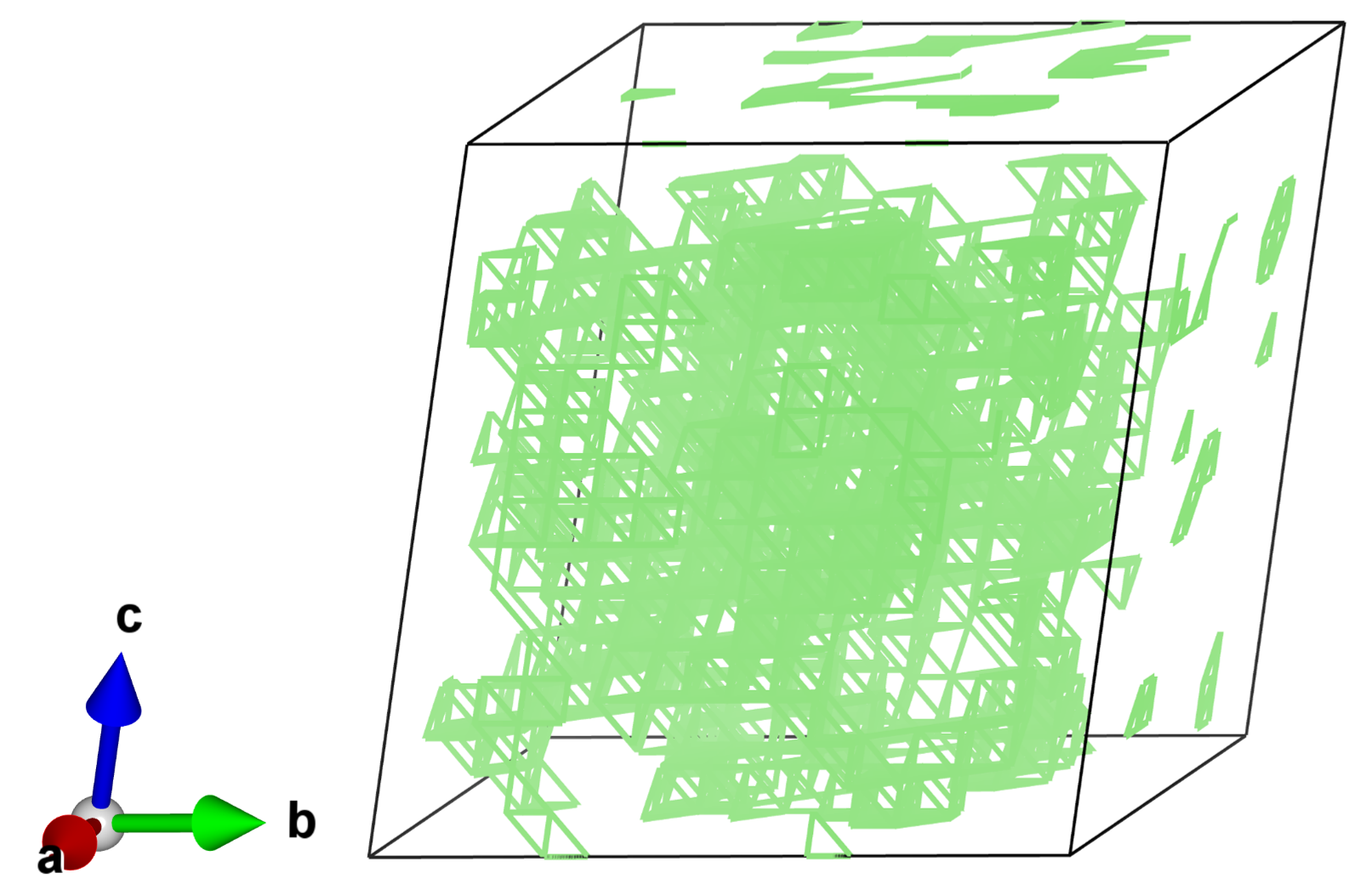


**Fig. S21.** Representative Monte Carlo simulated structures of Li_2_TiOF_2_ at 300 K (only the atoms contained in 0-TM are shown) 0-TM connected Li atoms are bridged with green lines


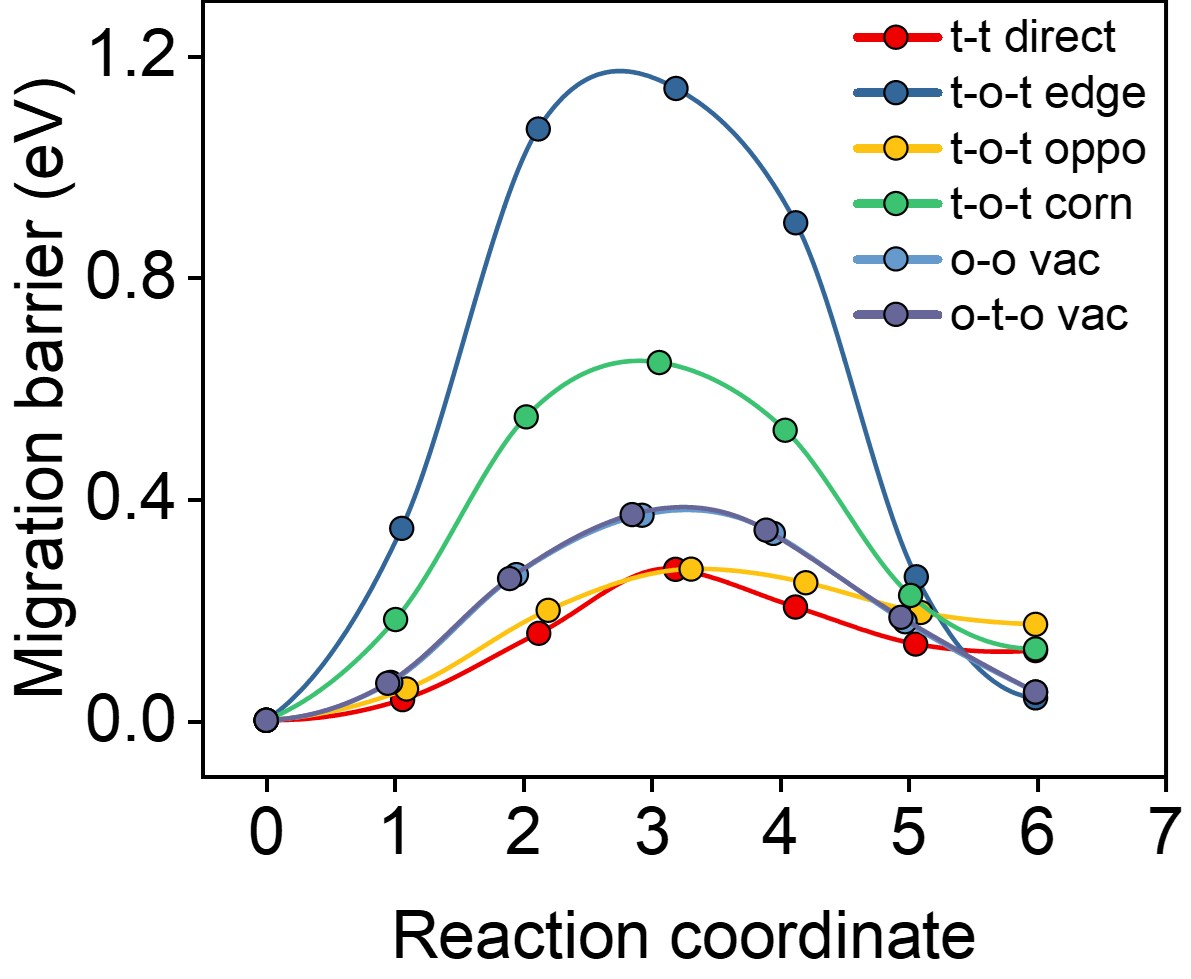


**Fig. S22** Li migration barriers in Li_2_TiOF_2_ SQoS structures


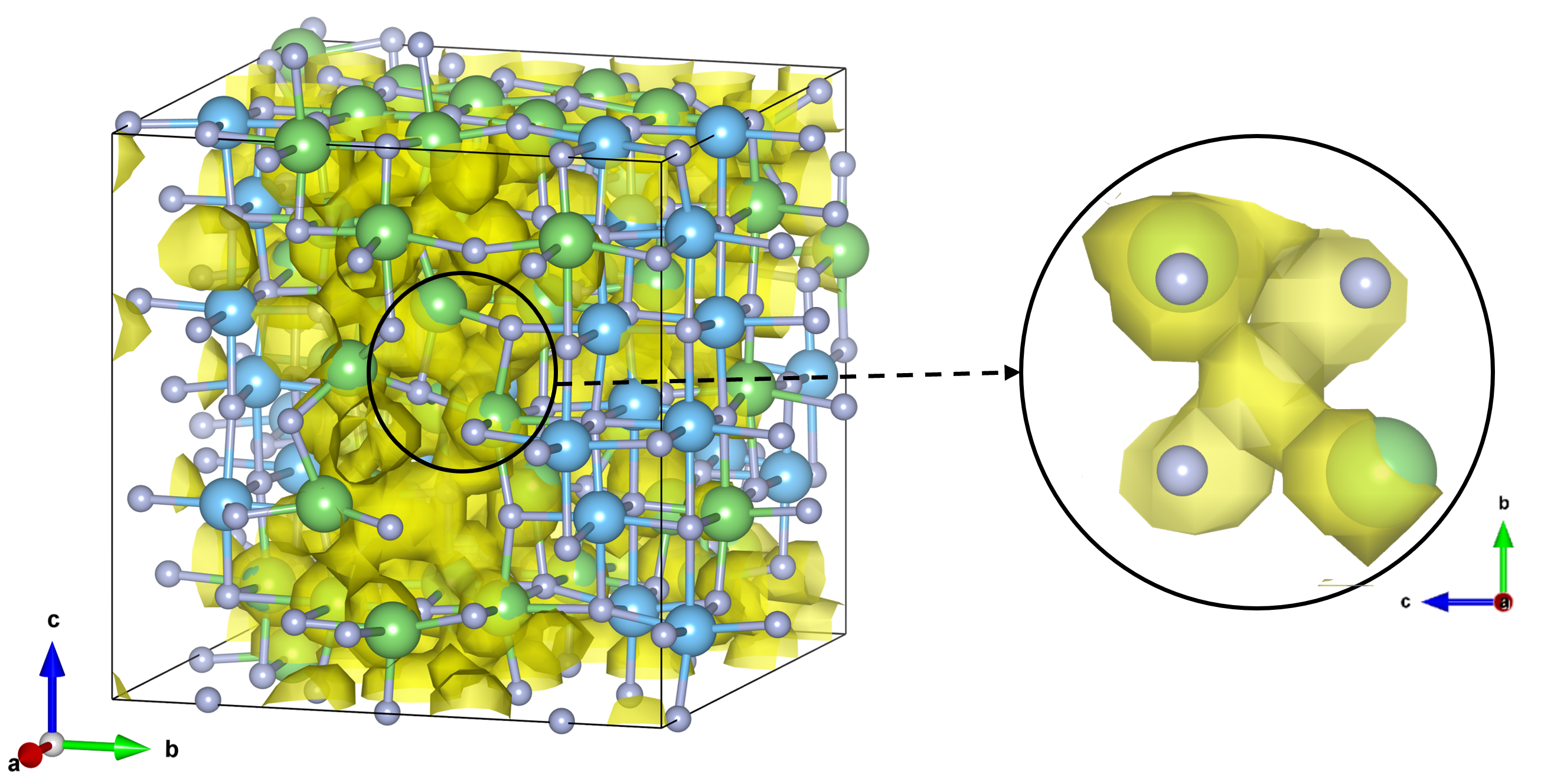


**Fig. S23** Li^+^ probability density of Li_1.5_TiOF_2_ at 1000 K from AIMD simulations


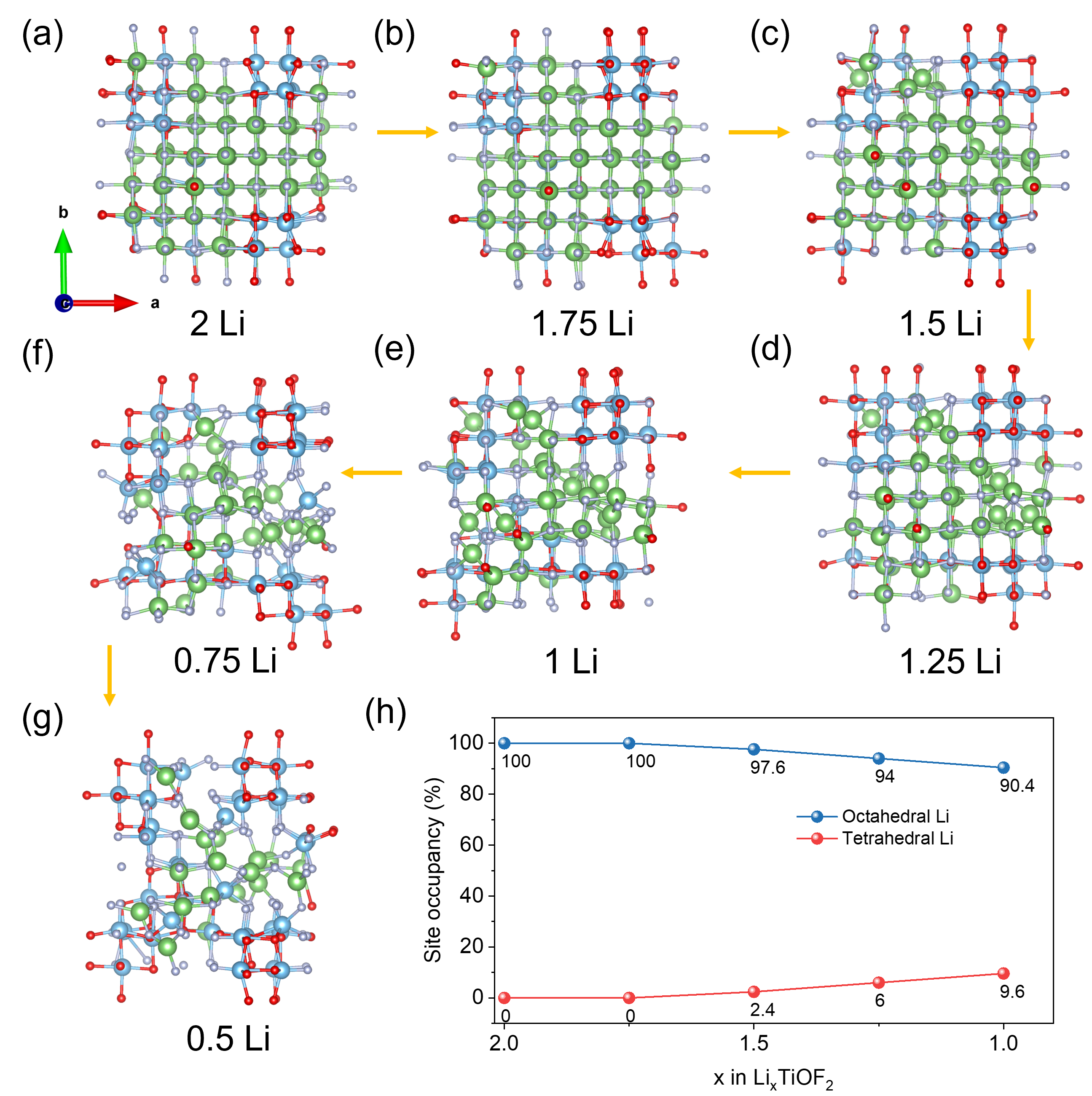


**Fig. S24** Atomic arrangement in the DRX structure during delithiation


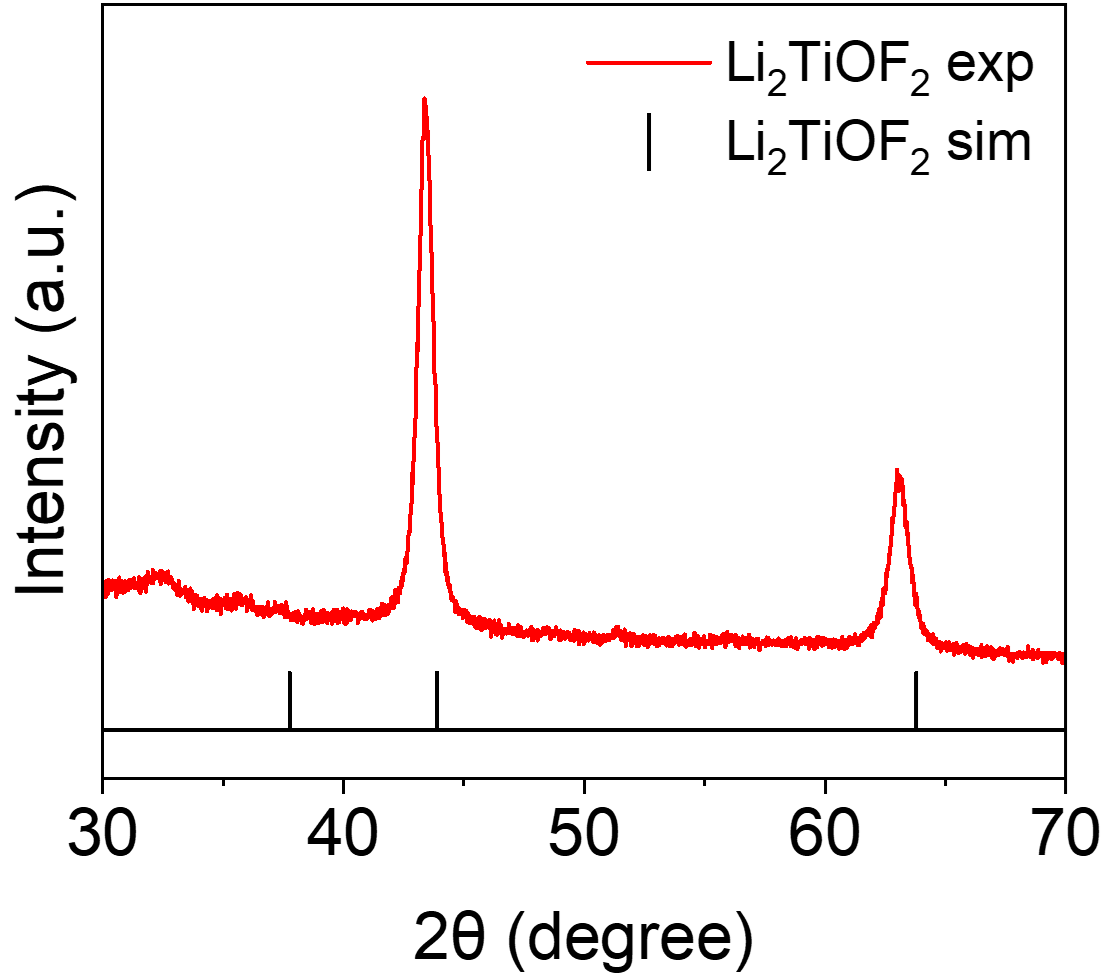


**Fig. S25** XRD patterns of Li_2_TiOF_2_. Red lines are experimental result, and black lines are calculated result by GSAS-II


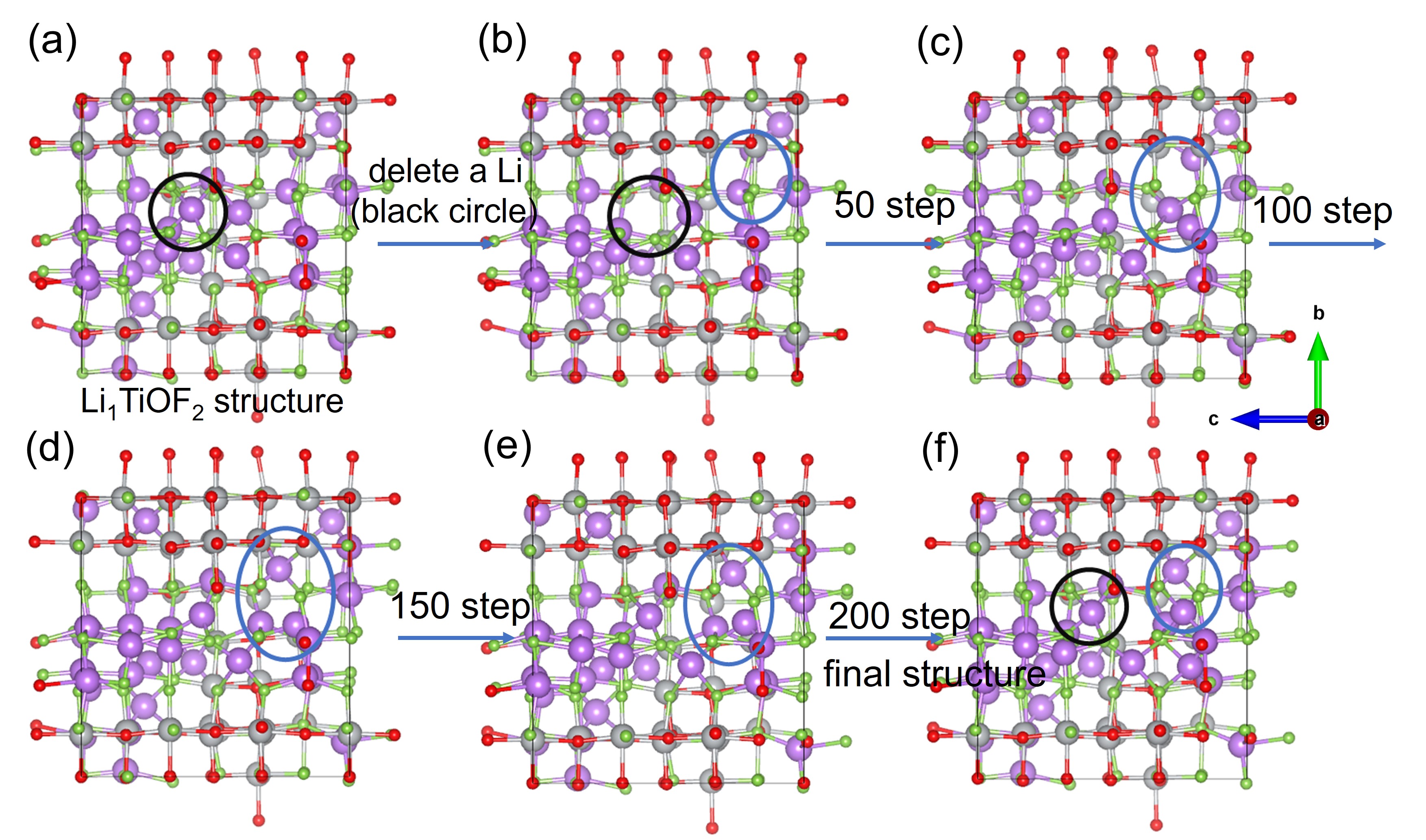


**Fig. S26** Location changes of atoms during Li_1_TiOF_2_ structure optimization at 300 K. (a) Representative SQoS structures of Li_1_TiOF_2_. (b) SQoS structure of Li_1_TiOF_2_ with one Li (black circle) removed. (c-e) A representative structure is taken every 50 optimization steps. (f) The final optimized structure

**Fig. S27** Galvanostatic discharge profiles at various current densities
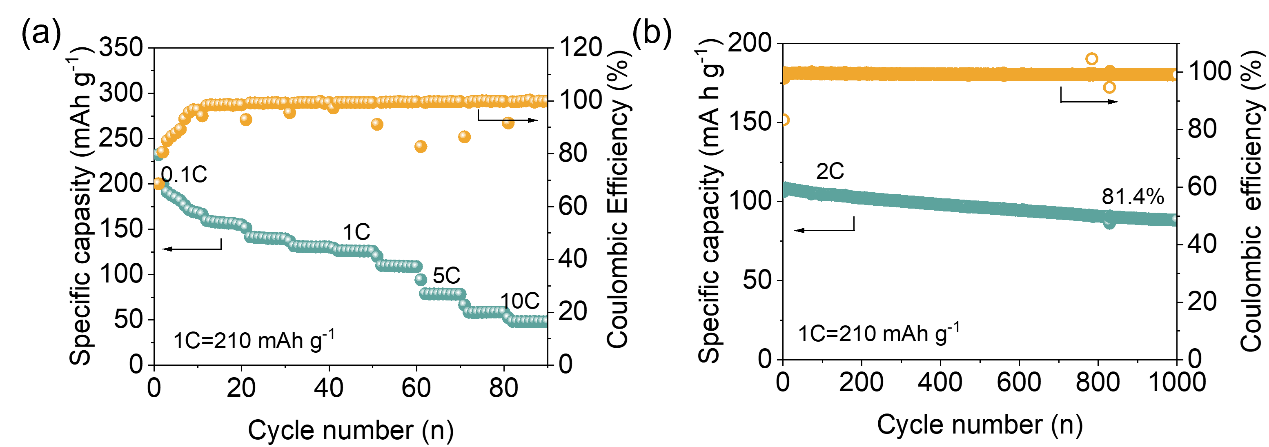


**Fig. S28.** (a) Rate capability from 0.1 to 10 C for DRX-LiₓTiOF₂//NMC811. (b) Long-term cycling stability for DRX-Li_x_TiOF_2_//NMC anodes at 2C


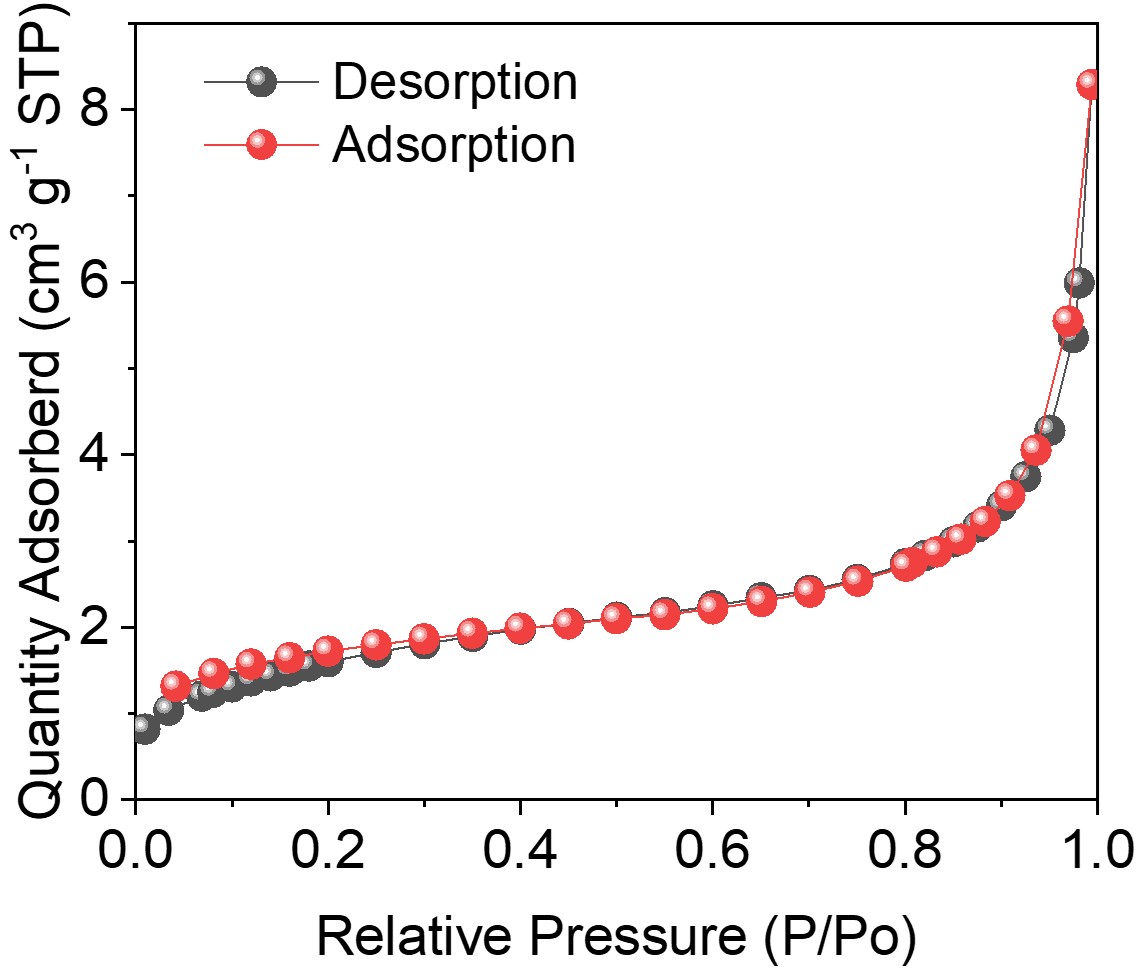


**Fig. S29** N_2_ adsorption–desorption isotherms of TiOF_2_

**Table S1** The results of ICP and XPS discharged to 0.1 V and charged to 2.0 V

| Test | Li/Ti atom |
| --- | --- |
| ICP D-0.1 V | 2.95 |
| ICP C-2 V | 1.89 |
| XPS D-0.1 V etch 1000s | 2.28 |
| XPS C-2.0 V etch 1000s | 1.19 |

**Table S2** Summary of electrochemical performances of materials

| Materials | Voltage window (V) | Maximum capacity (mAh g^-1^) |  | Cycles (n) |  | Capacity retention(%) | Refs. |
| --- | --- | --- | --- | --- | --- | --- | --- |
| TiO_2_(B) | 1-3 | 277 (0.25C) |  | 1000 (5C) |  | 91.8 | [S14] |
| Li_4_Ti_5_O_12_ | 1-2.5 | 170 (1C) |  | 500 (10C) |  | 86.6 | [S15] |
| Nb_2_O_5_ | 1.3-3 | 151 (0.1 A g^-1^) |  | 1000 (0.2 A g^-1^） |  | 65.3 | [S16] |
| Nb_18_W_16_O_93_ | 1-2.5 | 225 (C/5) |  | 750 (20C) |  | 92.3 | [S17] |
| Li_3_V_2_O_5_ | 0.1-2 | 207 (0.1 A g^-1^) |  | 1000 (2A g^-1^) |  | 93.3 | [S18] |
| H_2_Ti_6_O_13_ | 1-3.5 | 230 (0.1A g^-1^) |  | 1000 (1.5 A g^-1^ full cell) |  | 80.0 | [S19] |
| LiTiSiO_5_ | 0.1-3 | 258 (0.1 A g^-1^) |  | 2000 (2 A g^-1^ full cell) |  | 91.7 | [S20] |
| This work | 0.1-2 | 310 (0.1 A g^-1^) |  | 1000 (5 A g^-1^) |  | 74.0 |  |

**Supplementary References**

1. X. Zhao, Y. Tian, Z. Lun, Z. Cai, T. Chen et al., Design principles for zero-strain Li-ion cathodes. Joule **6**(7) 1654–1671 (2022). <https://doi.org/10.1016/j.joule.2022.05.018>
2. L. Huang, P. Zhong, Y. Ha, Z. Cai, Y.-W. Byeon et al., Optimizing Li-excess cation-disordered rocksalt cathode design through partial Li deficiency. Adv. Energy Mater. **13**(4) 2202345 (2023). <https://doi.org/10.1002/aenm.202202345>
3. M. Ångqvist, W.A. Muñoz, J.M. Rahm, E. Fransson, C. Durniak et al., ICET–a Python library for constructing and sampling alloy cluster expansions. Adv. Theory Simul. **2**(7) 1900015 (2019). <https://doi.org/10.1002/adts.201900015>
4. H. Ji, A. Urban, D.A. Kitchaev, D.-H. Kwon, N. Artrith et al., Hidden structural and chemical order controls lithium transport in cation-disordered oxides for rechargeable batteries. Nat. Commun. **10**, 592 (2019). <https://doi.org/10.1038/s41467-019-08490-w>
5. G. Kresse, J. Furthmüller, Efficiency of ab-initio total energy calculations for metals and semiconductors using a plane-wave basis set. Comput. Mater. Sci. **6**(1) 15–50 (1996). <https://doi.org/10.1016/0927-0256(96)00008-0>
6. J.W. Furness, A.D. Kaplan, J. Ning, J.P. Perdew, J. Sun, Accurate and numerically efficient r^2^SCAN meta-generalized gradient approximation. J. Phys. Chem. Lett. **11**(19) 8208–8215 (2020). <https://doi.org/10.1021/acs.jpclett.0c02405>
7. A. van de Walle, P. Tiwary, M. de Jong, D.L. Olmsted, M. Asta et al., Efficient stochastic generation of special quasirandom structures. Calphad **42**, 13–18 (2013). <https://doi.org/10.1016/j.calphad.2013.06.006>
8. Z. Cai, B. Ouyang, H.-M. Hau, T. Chen, R. Giovine et al., *In situ* formed partially disordered phases as earth-abundant Mn-rich cathode materials. Nat. Energy **9**(1) 27–36 (2024). <https://doi.org/10.1038/s41560-023-01375-9>
9. S.P. Ong, W.D. Richards, A. Jain, G. Hautier, M. Kocher et al., Python Materials Genomics (pymatgen): a robust, open-source Python library for materials analysis. Comput. Mater. Sci. **68**, 314–319 (2013). <https://doi.org/10.1016/j.commatsci.2012.10.028>
10. H. Liu, Z. Zhu, Q. Yan, S. Yu, X. He et al., A disordered rock salt anode for fast-charging lithium-ion batteries. Nature **585**(7823) 63–67 (2020). <https://doi.org/10.1038/s41586-020-2637-6>
11. S.Nosé, A unified formulation of the constant temperature molecular dynamics methods. J. Chem. Phys. **81**(1) 511–519 (1984). <https://doi.org/10.1063/1.447334>
12. G. Henkelman, B.P. Uberuaga, H. Jónsson, A climbing image nudged elastic band method for finding saddle points and minimum energy paths. J. Chem. Phys. **113**(22) 9901–9904 (2000). <https://doi.org/10.1063/1.1329672>
13. A. Van der Ven, G. Ceder, M. Asta, P.D. Tepesch, First-principles theory of ionic diffusion with nondilute carriers. Phys. Rev. B **64**(18) 184307 (2001). <https://doi.org/10.1103/physrevb.64.184307>
14. X. Li, G. Wu, X. Liu, W. Li, M. Li, Orderly integration of porous TiO_2_(B) nanosheets into bunchy hierarchical structure for high-rate and ultralong-lifespan lithium-ion batteries. Nano Energy **31**, 1–8 (2017). <https://doi.org/10.1016/j.nanoen.2016.11.002>
15. Y. Tian, Z.L. Wu, G.B. Xu, L.W. Yang, J.X. Zhong, Hetero-assembly of a Li_4_Ti_5_O_12_ nanosheet and multi-walled carbon nanotube nanocomposite for high-performance lithium and sodium ion batteries. RSC Adv. **7**(6) 3293–3301 (2017). <https://doi.org/10.1039/C6RA25651A>
16. Z. Hu, Q. He, Z. Liu, X. Liu, M. Qin et al., Facile formation of tetragonal-Nb_2_O_5_ microspheres for high-rate and stable lithium storage with high areal capacity. Sci. Bull. **65**(14) 1154–1162 (2020). <https://doi.org/10.1016/j.scib.2020.04.011>
17. K.J. Griffith, K.M. Wiaderek, G. Cibin, L.E. Marbella, C.P. Grey, Niobium tungsten oxides for high-rate lithium-ion energy storage. Nature **559**(7715) 556–563 (2018). <https://doi.org/10.1038/s41586-018-0347-0>
18. Y. Qin, H. Zhao, M. Hua, J. Gao, J. Lu et al., Pseudocapacitive lithium-rich disordered rock salt vanadium oxide with 3D lithium-ion transport pathways for high-performance lithium-ion capacitor. J. Power Sources **588**, 233722 (2023). <https://doi.org/10.1016/j.jpowsour.2023.233722>
19. Y. Wang, Z. Hong, M. Wei, Y. Xia, Layered H_2_Ti_6_O_13_-nanowires: a new promising pseudocapacitive material in non-aqueous electrolyte. Adv. Funct. Mater. **22**(24) 5185–5193 (2012). <https://doi.org/10.1002/adfm.201200766>
20. S. Wang, R. Wang, Y. Bian, D. Jin, Y. Zhang et al., *In-situ* encapsulation of pseudocapacitive Li_2_TiSiO5 nanoparticles into fibrous carbon framework for ultrafast and stable lithium storage. Nano Energy **55**, 173–181 (2019). <https://doi.org/10.1016/j.nanoen.2018.10.052>
